# Impact of construction and demolition wastes on the performance of sustainable LC^3^-based structural lightweight concrete

**DOI:** 10.1038/s41598-026-48036-x

**Published:** 2026-04-24

**Authors:** Esraa Kamal Sadik, Mohamed Abd Elrahman, Khaled A. Eltawil, Ahmed M. Tahwia

**Affiliations:** 1 Department of Structural Engineering, Faculty of Engineering, Mansoura University, Mansoura, 35511 Egypt; 2 Department of Civil Engineering, Faculty of Engineering, New Valley University, Kharga, Egypt

**Keywords:** LC^3^, Structural lightweight concrete, Crushed brick powder, Recycled concrete powder, Durability, Engineering, Environmental sciences, Materials science

## Abstract

This study explores the possibility of producing sustainable structural lightweight concrete (LWC) based on limestone calcined clay cement (LC³) using waste from construction and demolition. The main innovation is the dual substitution of waste-derived materials for traditional LC³ constituents: crushed brick powder (CBP) was used in place of metakaolin (MK), and recycled concrete powder (RCP) was used in place of limestone powder (LSP). To achieve lower densities, nine concrete mixtures were created using crushed brick as both fine and coarse aggregates in addition to an air-entraining agent. Flowability, dry density, ultrasonic pulse velocity, compressive strength, resistance to magnesium sulfate attack and high temperatures (200 and 400 degrees Celsius), water absorption, and porosity were all assessed through an extensive experimental program. With only a small drop in 28-day compressive strength (5–8%) and a slight increase in water absorption (10–12%), the results showed that CBP is a very promising substitute for MK. All mixtures met the structural LWC requirements of DIN EN 1045-1 (dry density of 1650–1850 kg/m³ and strength > 24 MPa), but substituting RCP for LSP resulted in a more noticeable decrease in 28-day compressive strength (15–20%) and an increase in water absorption (13–18%). Additionally, after 180 days of sulfate exposure, all LC³ systems showed very little mass loss (< 0.7%) and maintained over 80% of their residual strength at 400 °C. According to the study, CBP and RCP can effectively and sustainably replace MK and LSP in LC³-based LWC, allowing for a 60% reduction in clinker while preserving structural integrity and promoting waste valorization.

## Introduction

The cement industry significantly contributes to global greenhouse gas emissions, adversely impacting the environment. Numerous studies have been conducted to determine methods to reduce cement usage in construction projects without compromising the fundamental strength and durability of concrete. The use of supplemental cementitious materials (SCMs) as a substitute for cement is a recognized approach to achieve sustainability objectives^1^. In the manufacturing of low-carbon cement, kaolinite clays and limestone have emerged as major sources of SCMs^2^. A novel, economic, and eco-friendly binder has recently been developed utilizing a combination of limestone powder (LSP) and calcined clays such as metakaolin (MK), which is known as limestone calcined clay cement (LC^3^). This represents a promising approach to mitigate carbon emissions associated with the cement industry. This eco-friendly method enhances the properties of concrete while contributing to sustainability objectives^3^.

The growing amount of construction and demolition (C&D) waste especially bricks and concrete, is a big problem for the environment because it takes up valuable landfill space and can harm the quality of soil and water^4^. So, it’s very important for the construction industry to find ways to use these wastes in order to protect natural resources and reduce damage to the environment^5,6^. Tahwia et al^7^. illustrated that the inclusion of recycled concrete powder (RCP) or crushed brick powder (CBP) in geopolymer mortars can improve compressive strength and durability. Also, early studies of using RCP instead of LSP in LC³ systems have shown good results, with compressive strengths that are close to those of regular mixes^8^. The pozzolanic potential of fired clay brick powder in an LC³ blend has been verified, resulting in diminished porosity and reduced chloride ingress relative to conventional Portland cement.

The Marangu^9^ reported that fired rejected clay brick powder exhibited high pozzolanic activity when combined with LSP and clinker to produce an LC^3^binder. Compared with both Portland Pozzolanic cement and ordinary Portland cement (OPC), this system demonstrated reduced porosity and chloride ion diffusivity across various water-to-cement (w/c) ratios. Alghamdi et al^10^. used autoclaved aerated concrete waste as a partial replacement for natural sand to produce lightweight LC^3^ mortar. The results revealed a significant reduction in the thermal conductivity and density.

Lightweight concrete (LWC) has been extensively utilized in the construction industry for decades because of its low density and favourable thermal and sound insulation properties^11^. Using LWC helps reduce the dead weight of the structure, which lowers construction costs by allowing for smaller foundations and structural elements^12^. This type of concrete can have different density levels, making it suitable for a wide range of applications. It is generally divided into two types: cellular concrete, which is produced by introducing air voids into the mix, and lightweight aggregate concrete, which is manufactured from lightweight aggregates derived either from natural sources or produced from raw materials through industrial processes^13^. Compared with the use of conventional natural aggregates, the use of crushed brick (CB) as aggregates leads to a reduction in the overall weight of concrete because of their lower bulk density^4,13^. This makes CB a promising material for producing LWC. Atyia et al^14^. reported that when both fine and coarse aggregates were substituted with CB aggregates, the resulting concrete mixtures achieved dry densities of less than 2000 kg/m^3^, thereby meeting the classification criteria for lightweight aggregate concrete specified in DIN EN 1045–1^15^. Moreover, the utilization of CB as aggregate has been reported to improve the performance of concrete at elevated temperatures, as noted by Tahwia et al^16^. For air entrainment, small air bubbles are formed within the concrete and become part of the hardened cement matrix^17^. One common method to introduce these air bubbles is the addition of an air-entraining agent (AEA) during the mixing process. The use of AEA helps create uniformly distributed air voids throughout the concrete, which enhances its resistance to freezing and thawing cycles^18^. Al-Kroom et al^19^. reported that the addition of AEA to concrete decreased the dry density and improved the workability and thermal properties. Previous studies have investigated lightweight mortar and concrete incorporating conventional LC³, as summarized in Table 1.

**Table 1:** Summary of studies on lightweight mortar and concrete produced with conventional LC³.

REF	Type	Compressive strength (MPa)	Dry density (kg/m^3^)
Alghamdi et al^10^.	Mortar	13 − 29.8	≤ 1300
Hassan et al^20^.	Mortar	6.76 − 9.21	832 − 1210
Mukherjee et al^21^.	Concrete	8.23 − 30.74	≤ 2000
Alghamdi et al^22^.	Mortar	9.8 − 12.1	1200 − 1360

The aforementioned studies are mainly restricted to pastes, mortars, or non-structural applications, but they offer insightful information about the use of recycled powders in LC³ systems. For example, Tang et al^8^. did not extend the investigation to structural concrete or include lightweight aggregates; instead, they concentrated on the binder level. In a similar vein, Marangu^9^verified brick powder’s pozzolanic activity in LC³ pastes but did not discuss its use in conjunction with recycled concrete powder in a complete concrete matrix. Additionally, Alghamdi et al^10^. investigated lightweight mortars but did not look into using waste-derived materials to replace both MK and LSP. Therefore, the simultaneous use of CBP as a substitute for MK and RCP as a substitute for LSP in structural lightweight concrete is still unexplored, especially when combined with crushed brick aggregates and air-entraining agents. By creating a fully waste-based LC³-LWC system and assessing its performance in comparison to structural standards, this study seeks to close this crucial gap.

While looking for binders that will last, making LWC from recycled materials is another way to make construction more environmentally friendly. LWC lessens structural dead loads, which means smaller foundations and lower overall construction costs^11,12^. Using CB as a lightweight aggregate is very promising because it has a lower bulk density, which makes the concrete lighter overall^4,13^. Recent research has successfully demonstrated the production of structural LWC)by completely replacing natural aggregates with CB, achieving dry densities below 2000 kg/m³ in accordance with DIN EN 1045-1. Additionally, research on analogous sustainable composites, including those utilizing e-waste aggregates or nano-additives, highlights the necessity of comprehensive performance assessment, encompassing mechanical properties and durability across diverse conditions, to confirm their structural viability^16,19^. Nevertheless, the simultaneous utilization of C&D waste as a binder substitute (in LC³) and as lightweight aggregates, in conjunction with air-entraining agents to further diminish density, signifies a notable research deficiency. The authors are unaware of any previous study that has concurrently examined the dual substitution of MK and LSP with CBP and RCP, respectively, in LC³-based structural LWC.

Based on the abovementioned discussions, this research seeks to use of LC^3^ with waste-based binders in the production of LWC has not been widely studied. To the authors’ knowledge, no previous research has simultaneously employed CBP and RCP as dual waste-derived alternatives for both MK and LSP in structural LC³-based LWC. The main objective of this study is to close this gap and investigate the potential of sustainable LC^3^ systems for producing structural LWC by incorporating waste materials as alternatives to conventional binder constituents. CB were used as fine and coarse lightweight aggregates, in addition to adding AEA to produce lighter concrete. The effects of replacing MK and LSP with CBP and RCP, respectively, on the performance of the LWC were investigated. The fresh and hardened properties of the developed LWC were examined. Moreover, the physical and durability characteristics are evaluated via different experiments. In addition, TGA and SAI tests were conducted to assess the pozzolanic activity of the developed cementitious composites.

## Materials and mix proportions

### Materials

OPC (CEM I 42.5 N) manufactured by El-Arish Company, Egypt, that is compliant with BS EN 197-1^23^ was used in this study. It has a specific gravity and specific surface area of 3.15 g/cm^3^ and 3300 cm^2^/g, respectively. MK and LSP, according to BS EN 197-1, which have specific gravities of 2.4 and 2.8 g/cm³, respectively, were sourced from local suppliers in Helwan, Egypt.

**Fig. 1:**
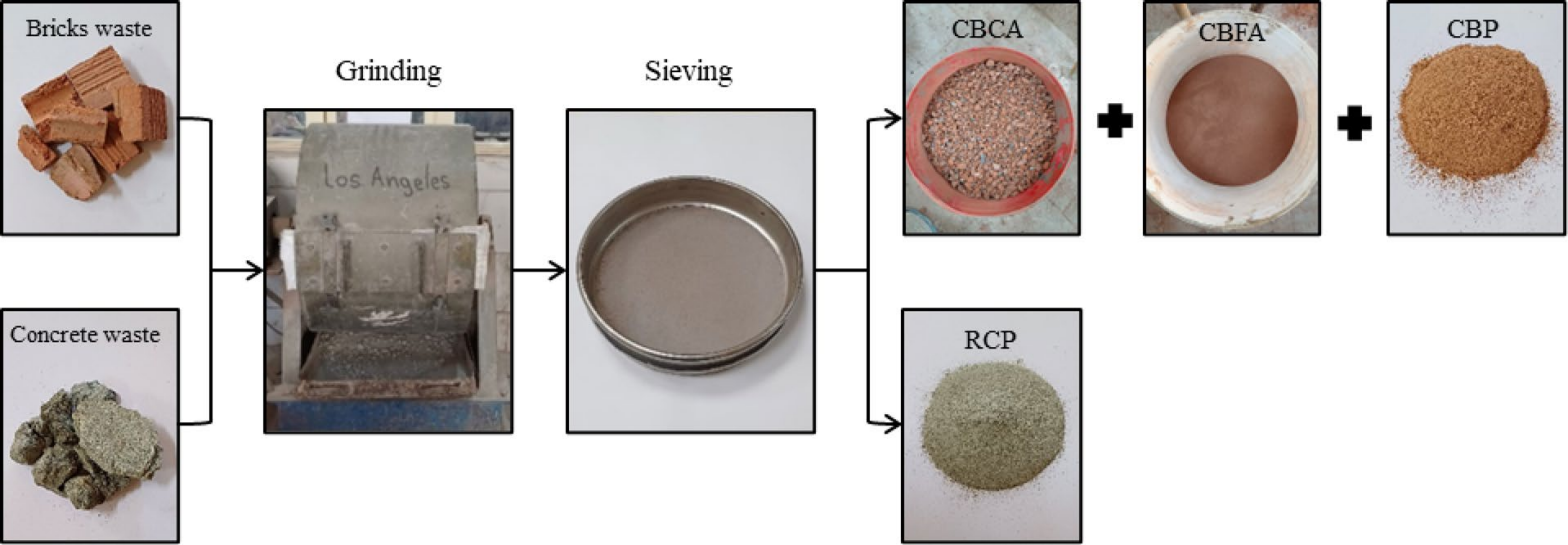
Preparation procedures for CBCA, CBFA, CBP, and RCP.

Figure 1 depicts the multi-step processing approach used to prepare aggregate and powder fractions from collected C&D trash. Initially, ancient clay bricks and concrete rubble collected from local demolition sites were crushed with a Los Angeles machine. The crushed material was then sieved to get the required particle size fractions. The percentage that passed through a 4.75 mm screen and was held on a 0.15 mm filter was referred to as crushed brick fine aggregate (CBFA). In contrast, the fraction kept on the 4.75 mm screen, with a nominal maximum size of 9.5 mm, was employed as crushed brick coarse aggregate (CBCA). To create the recycled powders (CBP and RCP), a part of the crushed material was ground in the Los Angeles machine until it passed through a 75 μm sieve. This resulted in a fine powder suitable for use as a binder component. Figure 1 shows a visual summary of these preparation steps.

**Fig. 2:**
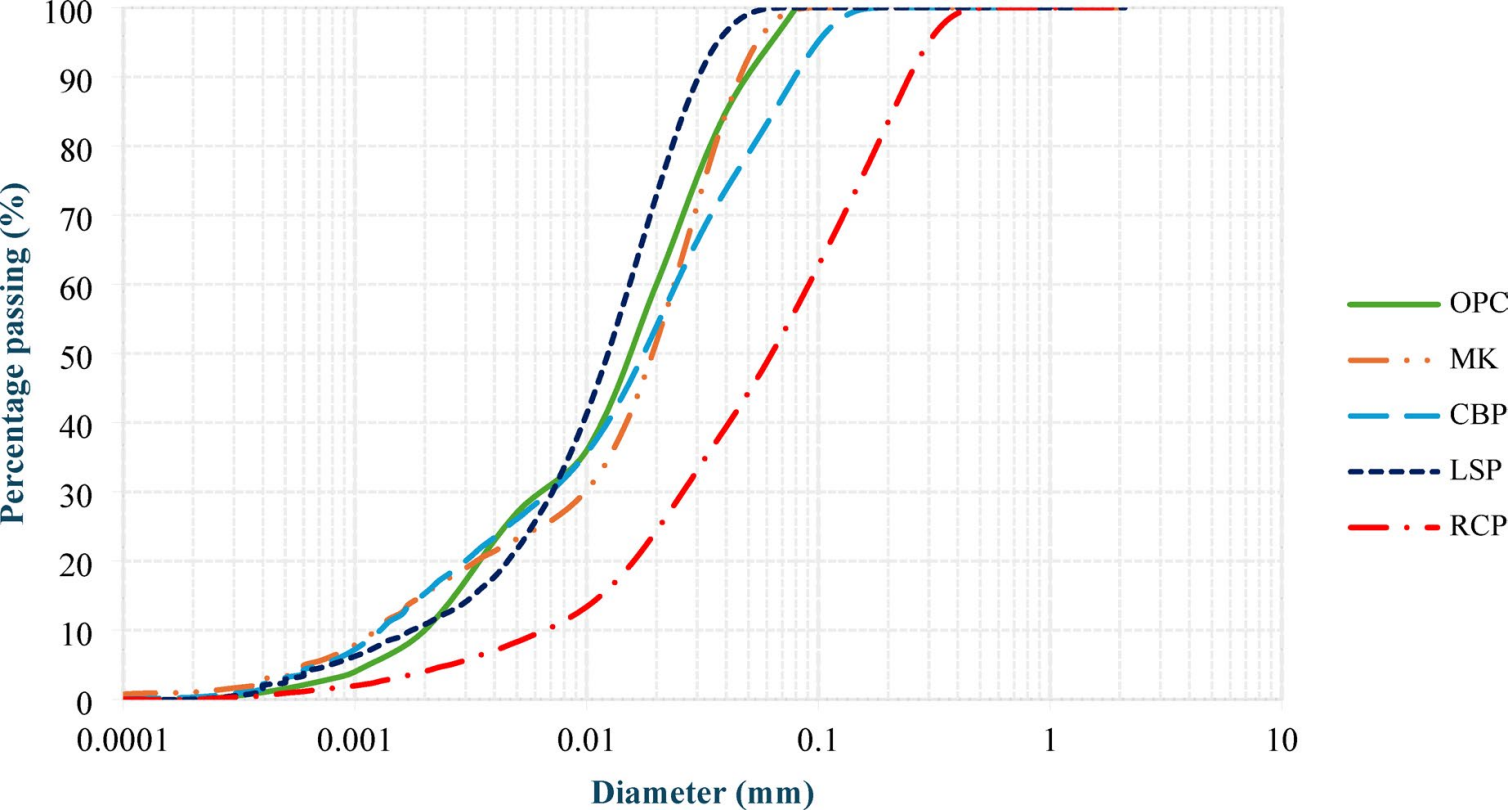
Measured particle size distributions of MK, CBP, LSP and RCP.

The specific gravities of CBFA and CBCA were found at 2.17 g/cm³. Given that aggregate water absorption has a substantial impact on both the fresh and hardened properties of lightweight concrete, this parameter was thoroughly assessed. The measured water absorption values were 11% for fine crushed brick aggregate and 9.5% for coarse fraction. CBP and RCP, with specific gravities of 2.25 g/cm³ and 2.6 g/cm³, replaced MK and LSP as binder components. The particle size distributions of these powders were validated by laser granulometry, as shown in Fig. 2.

**Fig. 3:**
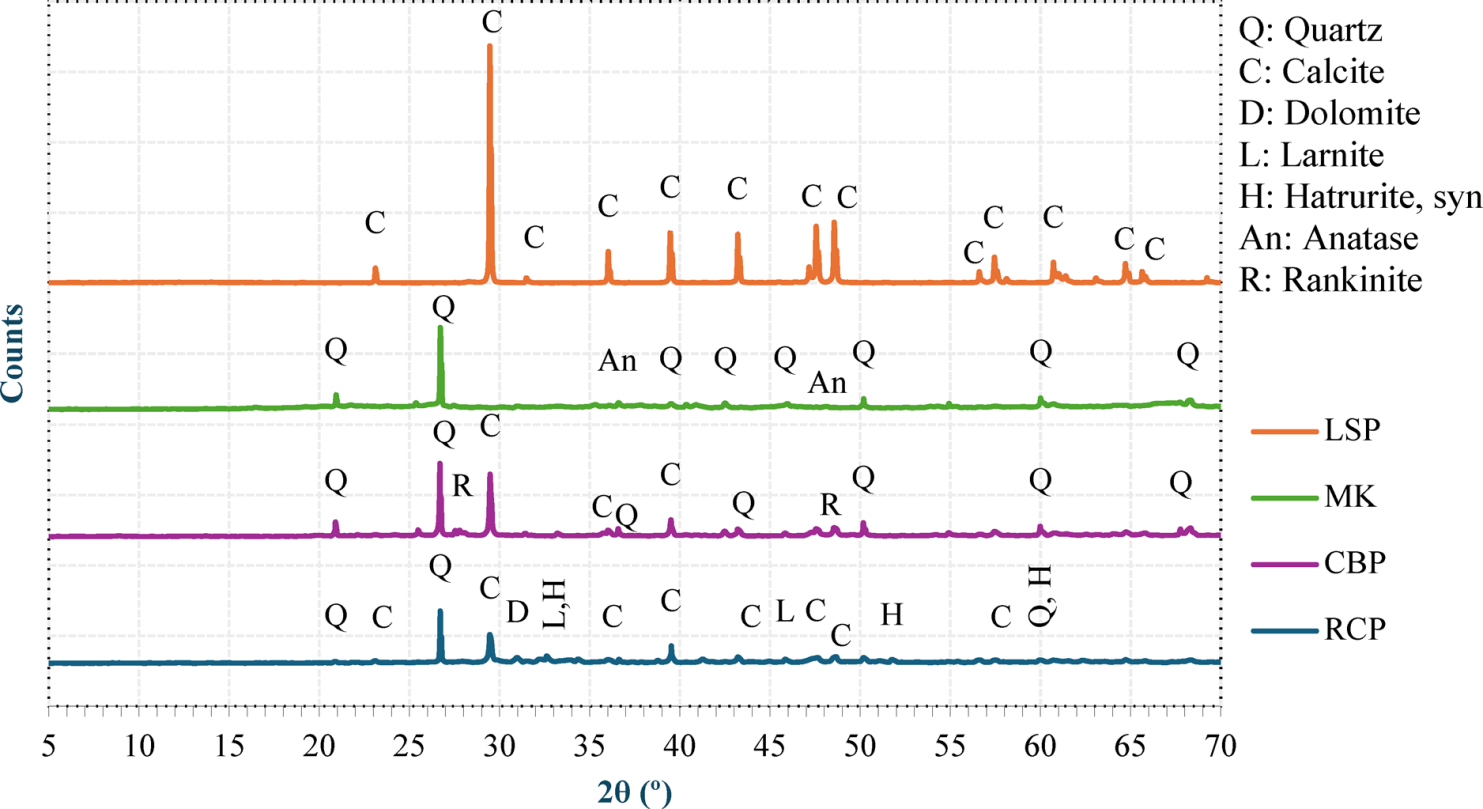
XRD analysis of the cementitious materials used.

**Fig. 4:**
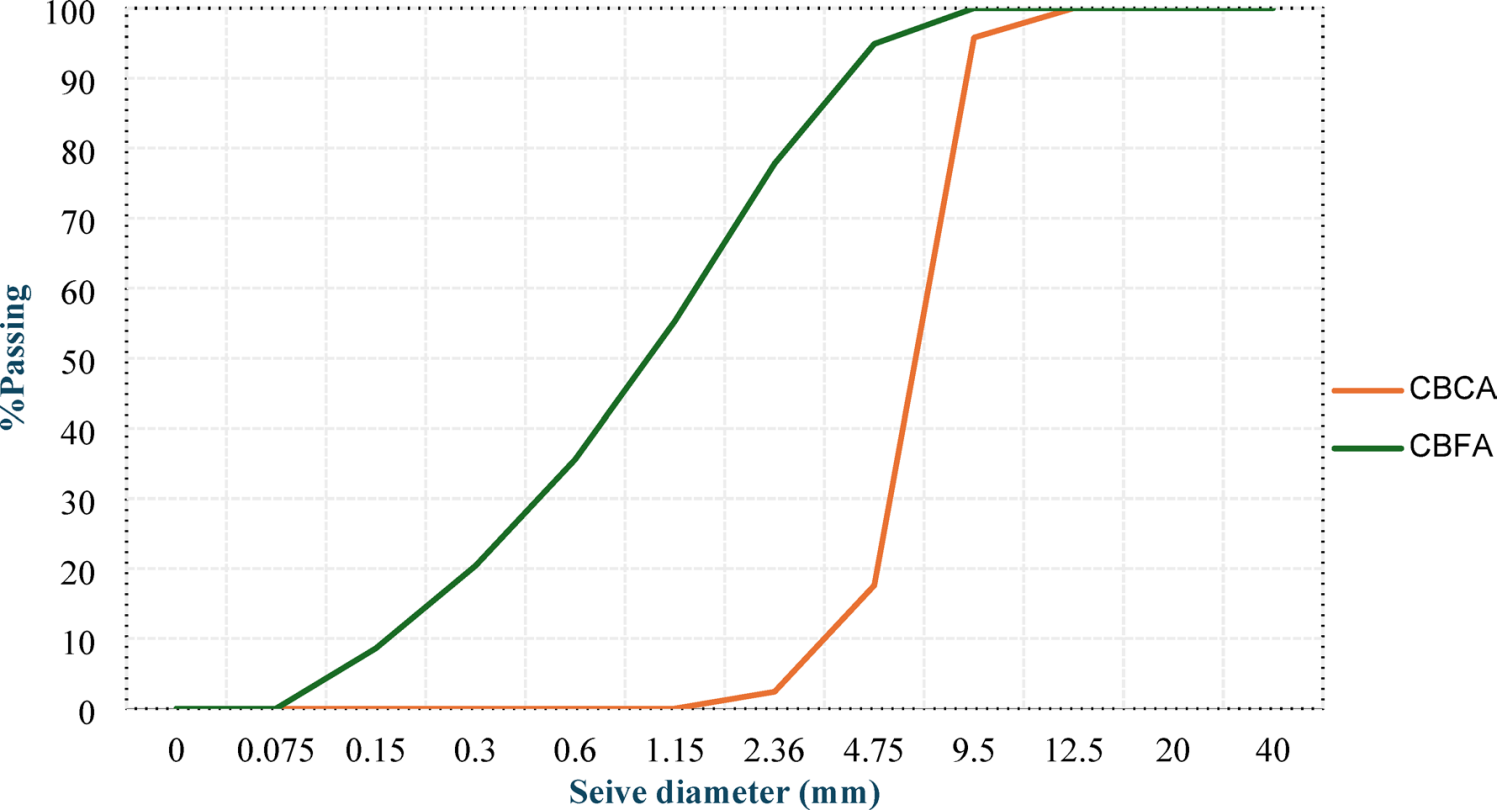
Sieve analysis of the used CBCA and CBFA.

The superplasticizer (SP), a high-range water-reducing admixture that conforms to ASTM C 494^24^, was incorporated to improve the workability of the concrete. A selective organic polymer-based AEA with a density of 1.02 kg/l according to ASTM C 260^25^ was employed to further decrease the concrete density. The particle size distributions of OPC, MK, CBP, LSP, and RCP were determined via laser granulometry and are depicted in Fig. 2. Figure 3 shows the XRD analysis, which showed that CBP and MK are similar in mineral composition (both contain a high amount of silica and alumina) and that RCP and LSP are similar (both contain a high amount of calcite). This mineralogical similarity is what makes it possible to investigate CBP and RCP as possible direct replacements for MK and LSP in the LC³ system. The chemical compositions of the investigated cementitious materials are shown in Table 2. Figure 4 shows the sieve analysis of used CBFA and CBCA.

**Table 2:** Chemical composition of the cementitious materials used.

Oxide composition (%)	OPC	MK	CBP	LSP	RCP
SiO_2_	21.15	52.40	51.80	0.90	40.96
Fe_2_O_3_	3.00	2.53	10.80	0.10	4.89
Al_2_O_3_	5.75	37.55	13.40	0.20	5.71
CaO	62.60	0.88	7.05	50.95	29.9
MgO	1.90	0.21	1.77	0.50	2.26
SO_3_	2.40	-	7.66	0.30	1.16
TiO_2_	0.30	-	1.82	0.01	0.26
LOI	1.70	0.98	1.74	42.00	9.59

## Mix proportions

**Fig. 5:**
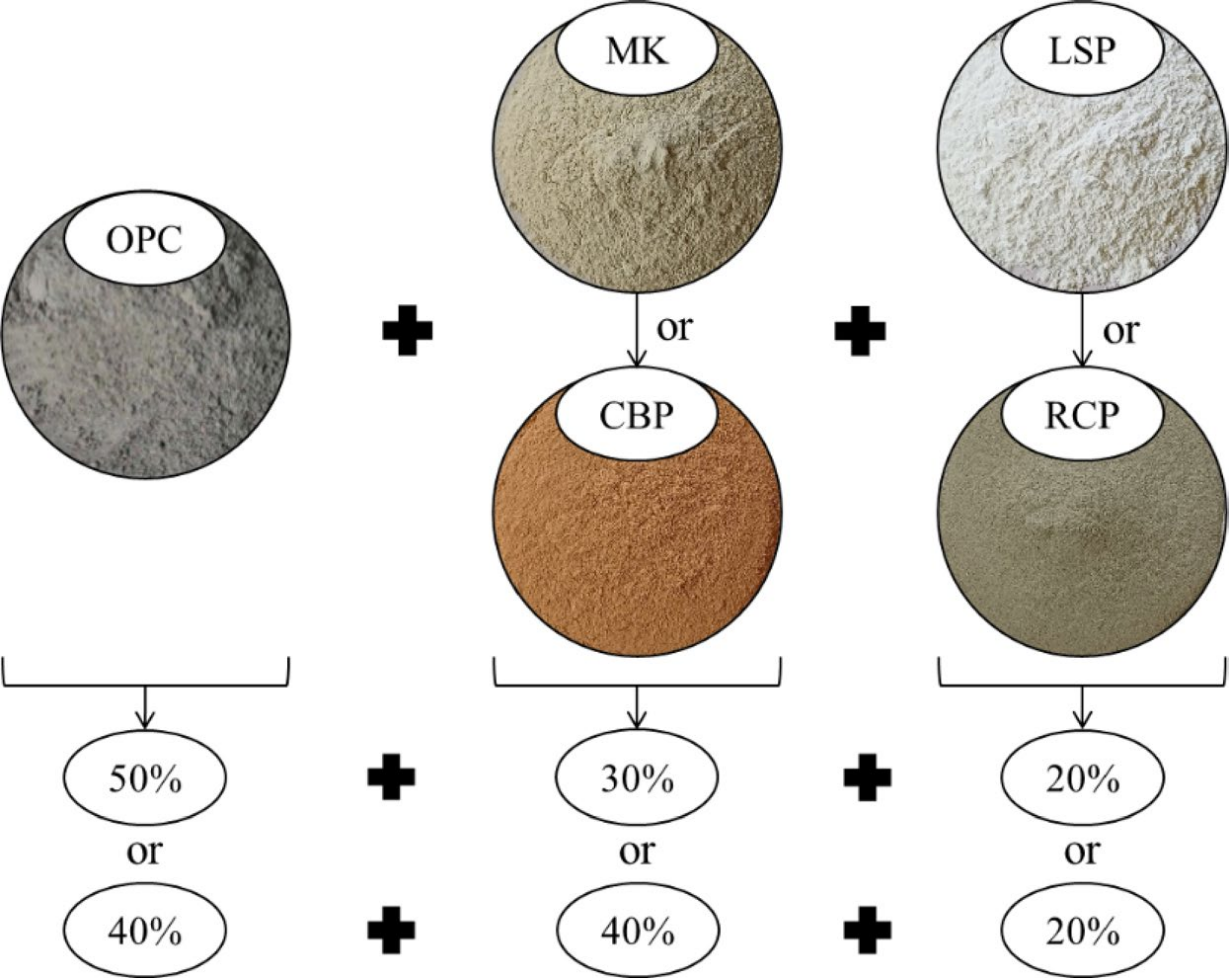
The proportionate distribution of components inside the binder of the concrete.

In this investigation, nine mixtures were designed, prepared and examined to achieve the objectives of the study. Table 3 presents the proportions of the concrete mixtures, which were designed according to the absolute volume method. The LC^3^ system is composed of three constituents: clinker (OPC), a calcined clay serving as the alumina source (MK or CBP), and a calcite source (LSP or RCP). The mixtures were categorized into two groups according to the type of calcite source: Group L, in which 20% LSP was incorporated by binder weight as the source of calcite, and Group R, in which RCP was incorporated as a replacement for LSP. Within each group, the binder was composed of clinker (40% and 50%), an alumina source (MK or CBP with 30% and 40%) and a 20% calcite source by weight, as suggested by previous studies^9,10^. AEA was added at a dosage of 0.25% of the binder mass to generate stable air bubbles, thus decreasing the dry density and improving the thermal characteristics, as documented by Al-Kroom et al^19^.. The control mixture (C) was prepared with 100% OPC without the addition of AEA. The binder used had a content of 450 kg/m^3^. CBFA and CBCA were used as fine and coarse aggregates, respectively. The CBFA: CBCA ratio was 1:1.5 by weight. All the mixtures had a consistent w/b ratio of 0.41, and the SP dose was varied from 1.5% to 2% of the binder mass to achieve appropriate slump flow values. To avoid poor workability, an additional amount of water equal to aggregate absorption was added to the mixing water. Figure 5 represents the proportionate distribution of components inside the binder of the concrete.

**Table 3:** Mix proportions of the LC^3^ concrete (kg/m^3^).

Mix Groups	Mixes	Binder					Aggregates		W	SP	AEA
		OPC	MK	CBP	LSP	RCP	CBFA	CBCA			
Control	C	450	-	-	-	-	544	816	185	6.75	-
L	MKL-30	225	135	-	90	-	544	816	185	6.75	1.125
	MKL-40	180	180	-	90	-	544	816	185	6.75	1.125
	CBL-30	225	-	135	90	-	544	816	185	6.75	1.125
	CBL-40	180	-	180	90	-	544	816	185	6.75	1.125
R	MKR-30	225	135	-	-	90	544	816	185	9	1.125
	MKR-40	180	180	-	-	90	544	816	185	9	1.125
	CBR-30	225	-	135	-	90	544	816	185	9	1.125
	CBR-40	180	-	180	-	90	544	816	185	9	1.125

## Mixing procedure

For all concrete mixtures, a consistent mixing method was used to make sure they were all the same and could be made again. Before mixing, the coarse and fine crushed brick aggregates were put in a saturated surface-dry (SSD) state so that they wouldn’t soak up the mixing water, which could make the workability worse. Furthermore, an additional amount of water equal to the total aggregate absorption was incorporated into the mixing water, ensuring that the effective w/b ratio remained constant and workability was not compromised. This approach is consistent with standard practices for lightweight aggregate concrete^14^.

A laboratory pan mixer was used to mix the ingredients. To make sure that the powders were evenly distributed, the dry binder parts (OPC, MK or CBP, and LSP or RCP) were first put in the mixer and mixed for 2 min. After that, the SSD fine and coarse aggregates were added and mixed with the binder for another two minutes. Then, the superplasticizer (SP) was added to the water that had been calculated to account for the w/b ratio and the water that had already been absorbed by the aggregates. As the mixer was running, this liquid mixture was slowly added to the dry materials. For 3 min, the mixing continued until the mixture was smooth and even. Finally, the air-entraining agent (AEA) was added, and the mixing continued for one more minute to make sure it was evenly spread throughout the fresh concrete. We measured the slump flow right after mixing. After that, the concrete was poured into the molds for each test, compacted with a vibrating table, and covered with plastic sheets to keep the moisture in. After 24 h, the specimens were taken out of their molds and put in water at room temperature (23 ± 2 °C) until they were ready to be tested.

## Experimental work

### Mixture preparation

Initially, coarse and fine aggregates were mixed in the mixer for 1 min, followed by the addition of the cementitious materials and further mixing for another 1 min. Subsequently, 2/3 of the mixing water was added, and the mixture was mixed for an additional 2 min. SP was mixed with the remaining water and then added to the mixer and mixed for 2 more min. For mixtures containing an AEA, it was added and mixed for an additional 2 min. The flowability of the fresh concrete was measured immediately after mixing via a slump flow test according to BS EN 12350-8^26^. Thereafter, the fresh concrete was cast in 150 × 150 × 150 mm^3^ and 70.5 × 70.5 × 70.5 mm^3^ cubical molds, which were covered with plastic foil and stored in a controlled chamber of temperature and relative humidity. After demolding, the specimens were cured in a water basin at room temperature until the designated testing age. The following tests were conducted: dry density, ultrasonic pulse velocity (UPV), compressive strength, elevated temperature resistance, magnesium sulfate resistance, water absorption and porosity.

### Testing

#### Reactivity of the materials used

**Thermogravimetric analysis (TGA)** was carried out to evaluate the pozzolanic activity of the investigated cementitious materials by measuring the mass loss associated with the dehydration of calcium hydroxide (CH). A decrease in the CH content reflects the degree of pozzolanic reaction between the tested material and the CH released during cement hydration. The test was performed in accordance with ASTM E 1131^27^. Five paste mixes were designed and tested according to ASTM C311^28^. The control mix (C) contained 100% OPC, whereas in the other mixes, 20% of the OPC was replaced by weight with each powder material. All mixes were prepared with a water-to-binder (w/b) ratio of 0.27 and adjusted to achieve a flow diameter of 20 ± 2 cm via flow table test according to ASTM C1437^29^. In parallel, the mixtures were cast into 50 mm cube molds, demolded after 24 h, and cured in water at room temperature for 28 days. Small samples were then extracted from the cube cores, ground into fine particles, and subjected to thermal analysis. The samples were heated at a rate of 20 °C/min to 800 °C, and the weight loss was recorded.

For the **strength activity index (SAI)**, five mortar mixtures were prepared following ASTM C311^28^ to assess the pozzolanic activity of the investigated cementitious materials. The control mix (C) consisted of 100% OPC, whereas in the other mixes, 20% of the cement was replaced by weight with each powder material. All mixes were prepared with a binder-to-sand ratio of 1:2.75 and a w/b ratio of 0.484. The mixtures were cast in 50 mm cube molds, demolded after 24 h, and cured in water at room temperature until testing. The compressive strength was measured at 7 and 28 days for three specimens of each age. The SAI was calculated via Eq. (1).

1 $$\:SAI\:=\:\frac{{\sigma\:}_{m}}{{\sigma\:}_{c}}\:\times\:100\:\%$$

where *SAI* is the strength activity index of a material (%), *σ*_*m*_ is the compressive strength of the sample containing the tested material (MPa), and *σ*_*c*_ is the compressive strength of the control sample (MPa).

#### Performance of the concrete mixtures

To achieve the objectives of this investigation, the fresh, physical, mechanical, thermal, and durability properties of the developed LWC were systematically evaluated. The fresh properties were assessed via the **slump flow test** to determine the flowability of the fresh LC^3^ LWC mixtures. The test was performed with a steel cone measuring 300 mm in height, a 200 mm base diameter, and a 100 mm top diameter. The test was conducted in accordance with BS EN 12350-8^26^. For the physical properties, the **dry density** of three specimens (150 × 150 × 150 mm^3^) of each mixture was measured at 28 days according to EN 12390-7^30^. The specimens were dried at a controlled temperature of 105 ± 5 °C until a constant weight was achieved. The dry density was then calculated by dividing the dry mass by the specimen volume. Additionally, the ultrasonic pulse velocity **(UPV)** was measured for three specimens (70.5 × 70.5 × 70.5 mm^3^) of each mixture at 28 days in accordance with ASTM C597^31^. The UPV value was determined by dividing the specimen’s length by the measured pulse transit time. For the mechanical properties, **compressive strength** development was monitored at 7, 28, and 90 days via three specimens (70.5 × 70.5 × 70.5 mm^3^) per mixture at each age, as specified in ASTM C109^32^.

For the thermal properties, the resistance of the concrete to elevated temperatures was evaluated following guidelines adapted from previous studies^16,33^. Six 28-day-old specimens (70.5 × 70.5 × 70.5 mm³) for each mixture were dried at 105 ± 5 °C to a constant mass. Three specimens were subsequently heated to 200 °C, and the remaining three specimens were heated to 400 °C for 3 h at a heating rate of 5 °C/min and a cooling rate of 2 °C/min, using an electric furnace. After cooling to room temperature, the relative residual mass and residual compressive strength were determined. The relative residual mass for each specimen was calculated via Eq. (2).

2 $$\:{M}_{r}\:=\:\frac{{M}_{f}}{{M}_{^\circ\:}}\:\times\:100\:\%$$

where *M*_*r*_ is the relative residual mass for a specimen after heat exposure (%), *M*_*f*_ is the specimen mass after heat exposure (g), and *M*_0_ is the specimen mass before heat exposure (g).

Several tests were conducted to evaluate the durability properties of the concrete. The impact of **magnesium sulfate** on the LC^3^ concrete specimens was investigated by immersing six 28-day-old specimens (70.5 × 70.5 × 70.5 mm^3^) in a 5% magnesium sulfate solution diluted with water, per ASTM C1012^34^. Three specimens were immersed for 90 days, whereas the other three specimens were immersed for 180 days. The exposure regime consisted of cyclic immersion in the solution for 14 days followed by 14 days of air drying at room temperature, and this cycle was repeated until the end of the specified exposure periods. Upon completion of the exposure, the mass change and compressive strength were evaluated. The mass change for each specimen was calculated via Eq. (3).

3 $$\:\varDelta\:M\:=\:\frac{{M}_{f}-{M}_{^\circ\:}}{{M}_{^\circ\:}}\:\times\:100\:\%$$

where *∆M* is the mass change of a specimen after magnesium sulfate exposure (%), *M*_*f*_ is the specimen mass after magnesium sulfate exposure (g), and *M*^0^ is the specimen mass before magnesium sulfate exposure (g).

The **water absorption and porosity** of three specimens (70.5 × 70.5 × 70.5 mm^3^) of each mixture were determined at 28 days in accordance with ASTM C642^35^. The specimens were oven-dried at 105 ± 5 °C for 24 h, after which the mass of the oven-dried specimens was measured. The specimens were subsequently immersed in tap water for another 24 h, after which the immersed mass was measured. Finally, the surface water of the saturated specimens was removed via a towel, after which the saturated surface-dry (SSD) mass was measured. The water absorption and porosity were calculated via Eq. (4) and Eq. (5), respectively.

4 $$\:WA\:=\:\frac{C-A}{A}\:\times\:100\:\%$$

5 $$\:P\:=\:\frac{C-A}{C-B}\:\times\:100\:\%$$

where *WA* is the water absorption of a specimen (%), *P* is the porosity of a specimen (%), *A* is the oven-dried mass of a specimen (g), *B* is the immersed mass of a specimen (g), and *C* is the SSD mass of a specimen (g). In all tests, three specimens were examined, and the average value was recorded.

## Results and Discussion

### Reactivity of the materials used

#### Thermogravimetric analysis (TGA)

**Fig. 6:**
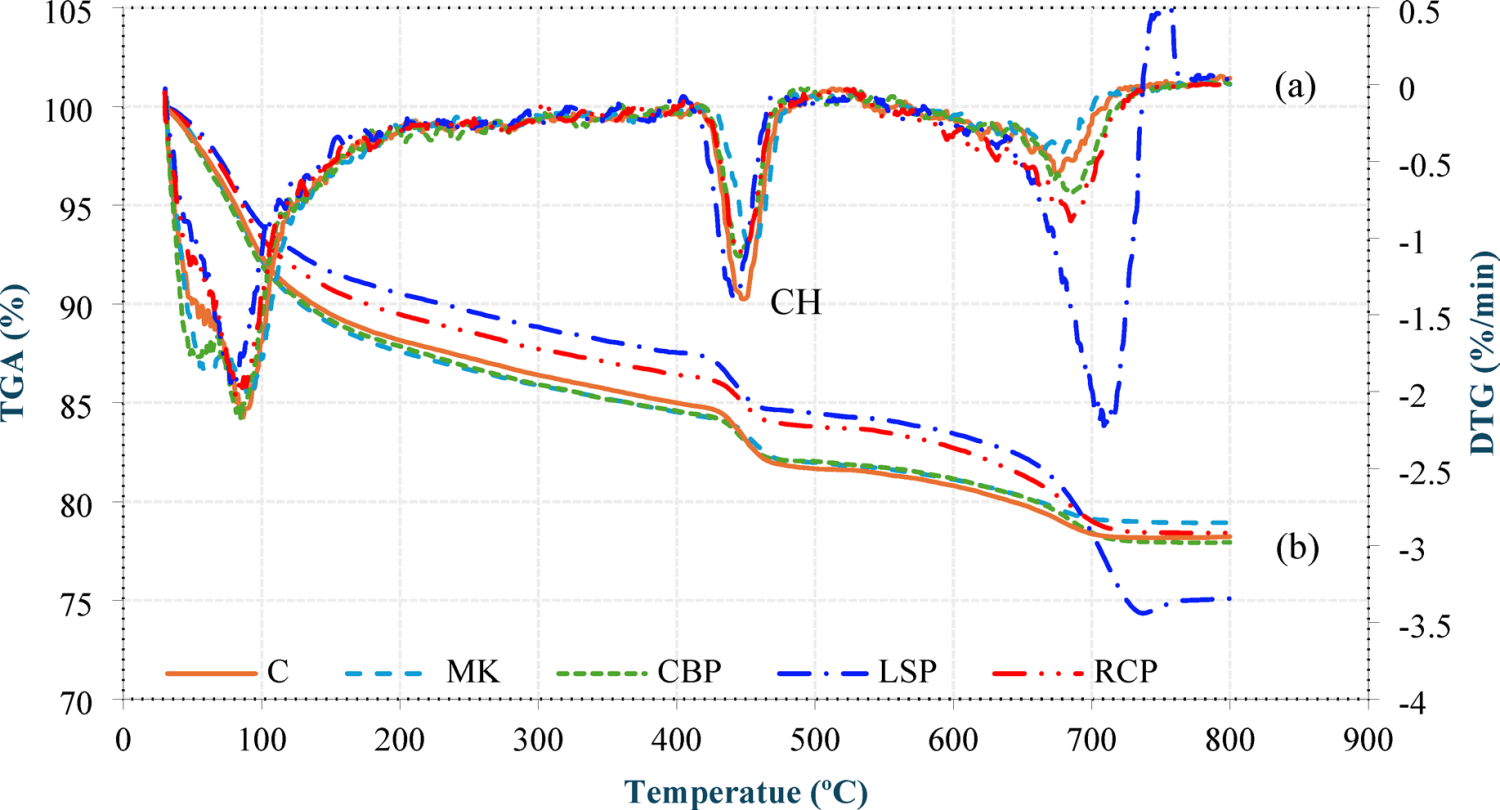
TGA (a) and DTG (b) analyses of the pastes of the tested cementitious materials at 28 days.

The TGA and DTG results are presented in Fig. 6. The dehydration of CH was observed between 400 °C and 500 °C. A lower mass loss indicates greater pozzolanic activity, as it reflects the reduced amount of CH resulting from its consumption during the pozzolanic reaction. The mass loss of CBP was 2.347%, whereas it was 2.335% for MK, indicating that CBP consumed slightly less CH than did MK. These findings indicate that, compared with MK, CBP has comparable or slightly lower pozzolanic activity. The mass loss of RCP was 2.416%, whereas it was 2.93% for LSP, indicating that RCP consumed more CH than LSP did. This suggests a potential pozzolanic activity of RCP, though limited in magnitude^36^.

#### Strength activity index (SAI)

**Fig. 7:**
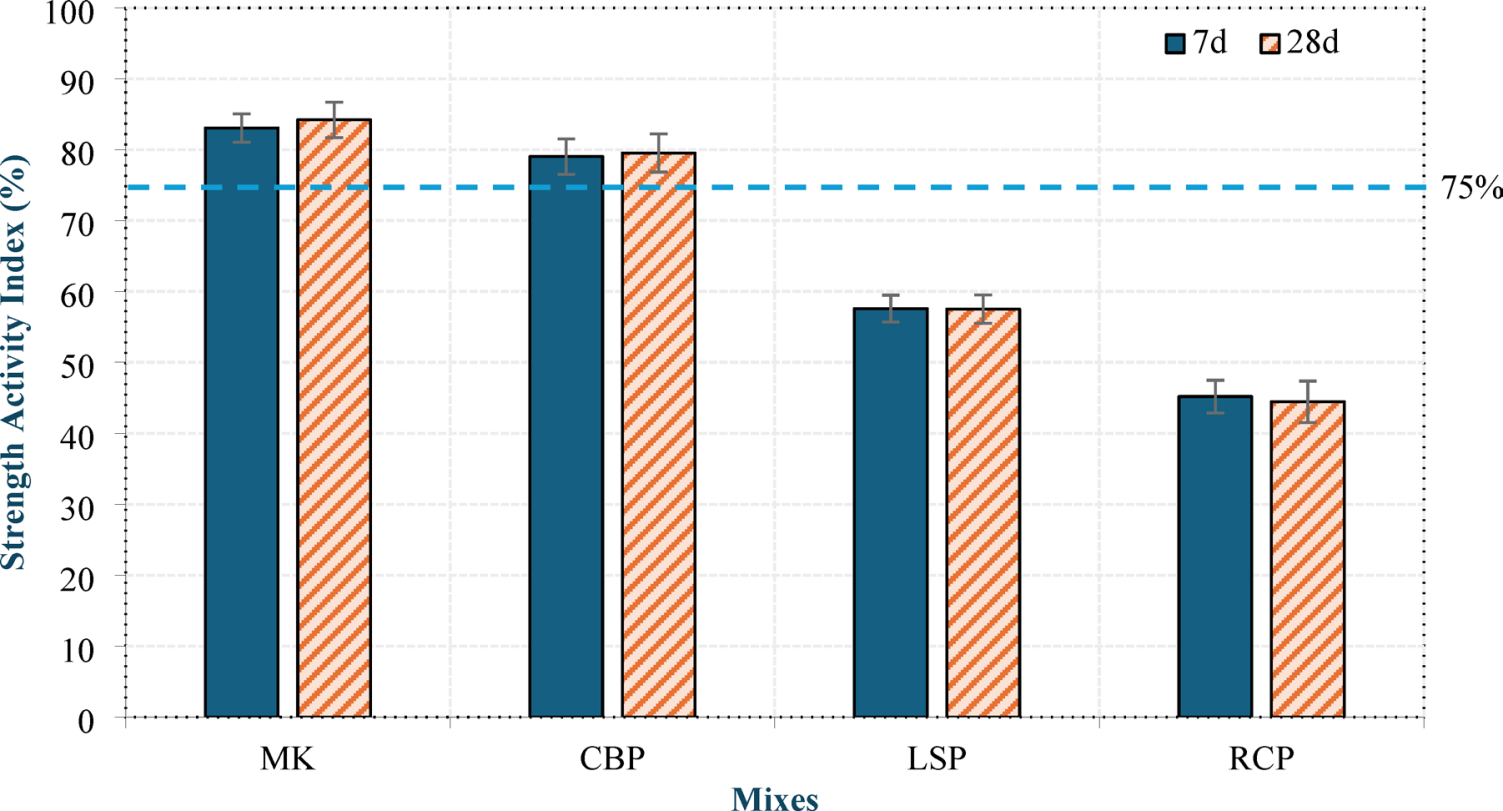
SAI values of the raw material mortars after 7 and 28 days.

The results of the SAI are presented in Fig. 7. The results show that MK and CBP achieved SAI values higher than 75% at both 7 and 28 days, fulfilling the requirements of ASTM C 618^37^, and thus can be classified as pozzolanic materials. The SAI values for MK were 83.06% and 84.21% at 7 and 28 days, respectively, whereas they were 79.03% and 79.53% for CBP at 7 and 28 days, respectively. This indicates that, compared with MK, CBP has slightly lower pozzolanic activity, which is consistent with the TGA results. In contrast, both RCP and LSP presented SAI values less than 75%, indicating low pozzolanic activity for RCP and confirming the non-pozzolanic nature of LSP. Nevertheless, LSP showed higher SAI values than those of RCP, although it consumes lower CH. The SAI values for LSP were 57.58% and 57.5% at 7 and 28 days, respectively, whereas they were 45.16% and 44.44% for RCP at 7 and 28 days, respectively. This can be attributed to the filler effect and finer particle size of LSP, which improved the packing density of the mixture^38^.

### Performance of the concrete mixtures

#### Slump flow test

**Fig. 8:**
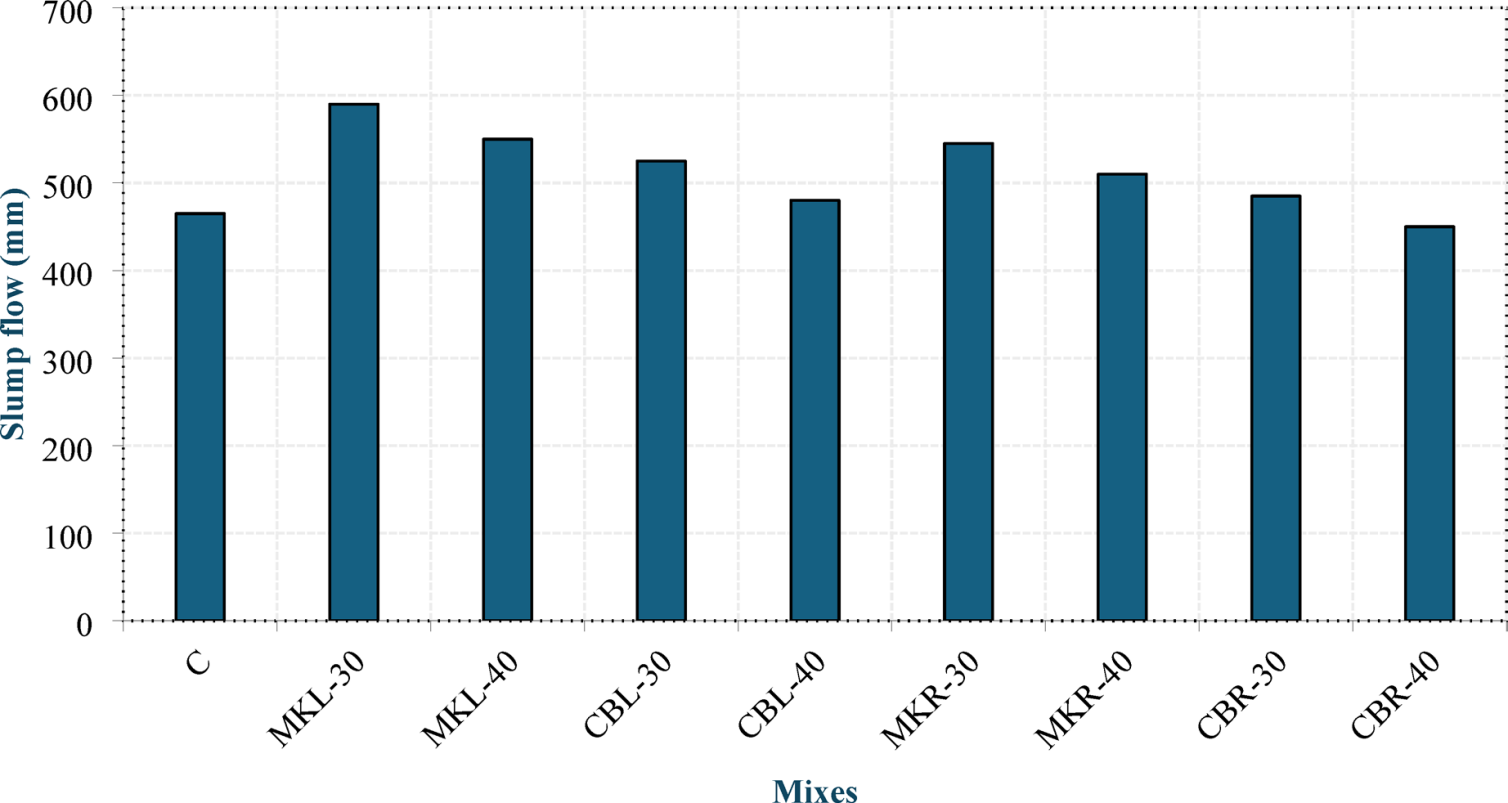
Slump flow values of fresh LC^3^ LWC.

Figure 8shows the slump flow values of the produced concrete. Compared with the control mixture (C), the incorporation of LSP improved the flowability. The increase in flow diameter ranged from approximately 3% to 27%. This enhancement can be attributed to the morphology and characteristics of LSP, which enhances flowability, as well as the synergistic effect between LSP and calcined clay, which is known to enhance the workability of LC³ systems, as reported in the review study by Barbhuiya et al^3^.. However, increasing the replacement level of either MK or CBP led to a reduction in the slump flow diameter. The incorporation of 40% MK resulted in a reduction in the flow diameter by 6.78% and 6.42% in group L and group R, respectively, compared with mixes containing 30% MK. Additionally, the incorporation of 40% CBP resulted in a reduction in the flow diameter by 8.57% and 7.22% in group L and group R, respectively, compared with mixes containing 30% CBP. This can be explained by their high specific surface area and internal porosity, which increase the overall water demand and reduce the free water available for flow. In addition, the fine clay-based particles tend to form flocculated structures that retain part of the mixing water. Their negatively charged surfaces also adsorb large amounts of Ca²⁺ ions, which affects the adsorption of PCE-based SP, thereby reducing their dispersing efficiency, as reported by Li et al^39^.. These combined effects ultimately lead to reduced flowability. Youssf et al^40^. reported that the workability of fresh mortar decreases with increasing MK content. Mixtures incorporating CBP exhibited lower flowability than those with MK at the same replacement level. The reduction in flow diameter was approximately 11–13% in group L and 11–12% in group R. This reduction can be attributed to the greater water absorption of CBP, which results from its porous structure and irregular particle shape that tends to interlock and retain more mixing water^14^. As shown in the figure, the group R mixes presented slightly lower flow diameters than those of group L mixes, despite the higher dosage of SP incorporated into the group R mixes. The reduction in flow diameter ranged from 6% to 8%. This can be attributed to the uneven morphology of the RCP particles, which increases the friction between the particles and decreases the flowability of the concrete. This is in line with the findings reported by Rocha et al^41^.. Tang et al^8^. also reported that, compared with LSP, the incorporation of RCP increases the viscosity and decreases the workability of the system.

#### Dry density

**Fig. 9:**
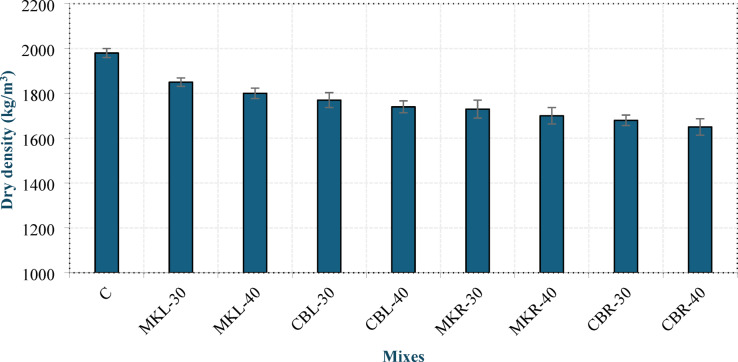
Dry density of LC^3^ LWC at 28 days.

The measured dry density of the produced concrete at 28 days is presented in Fig. 9. It is obvious that mix C has a dry density below 2000 kg/m^3^, indicating that CBFA and CBCA can be used to produce lightweight aggregate concrete according to DIN EN 1045-1 ^15^. This result agrees with that of Atyia et al^14^.. Increasing the replacement level of either MK or CBP led to a minimal decrease in the dry density of the concrete. Compared with the addition of 30% MK, the incorporation of 40% MK reduced the dry density by 2.7% and 1.73% in groups L and R, respectively. Similarly, incorporating 40% CBP resulted in reductions of 1.69% and 1.79% in groups L and R, respectively, relative to their corresponding 30% CBP mixes. This can be explained by the inherently lower specific gravity of both MK and CBP (2.4 g/cm^3^ and 2.25 g/cm^3^, respectively) than that of OPC (3.15 g/cm^3^), which contributes to the decrease in total concrete density when the replacement level is increased. Mixes incorporating CBP exhibited lower dry densities than those containing MK at the same replacement level. In group L, the reduction was 4.32% at 30% replacement and 3.33% at 40% replacement, whereas in group R, it was 2.89% and 2.94% at 30% and 40% replacement, respectively. This can be attributed to the high internal porosity of CBP, as reported by Atyia et al^14^.. As illustrated in the figure, the dry densities of the R mixtures were lower than those of the L mixtures, with a reduction ranging between 5.08% and 6.49%. This may be due to the porous structure and adhered mortar of the RCP particles^42^, in addition to their irregular morphology, which tends to entrap additional air. In contrast, the denser matrix and higher bulk density observed in group L mixtures can be attributed to the well-documented filler effect of LSP, which is known to improve particle packing and provide nucleation sites for hydration products, as extensively reported in the literature^38^.

#### Ultrasonic pulse velocity (UPV)

**Fig. 10:**
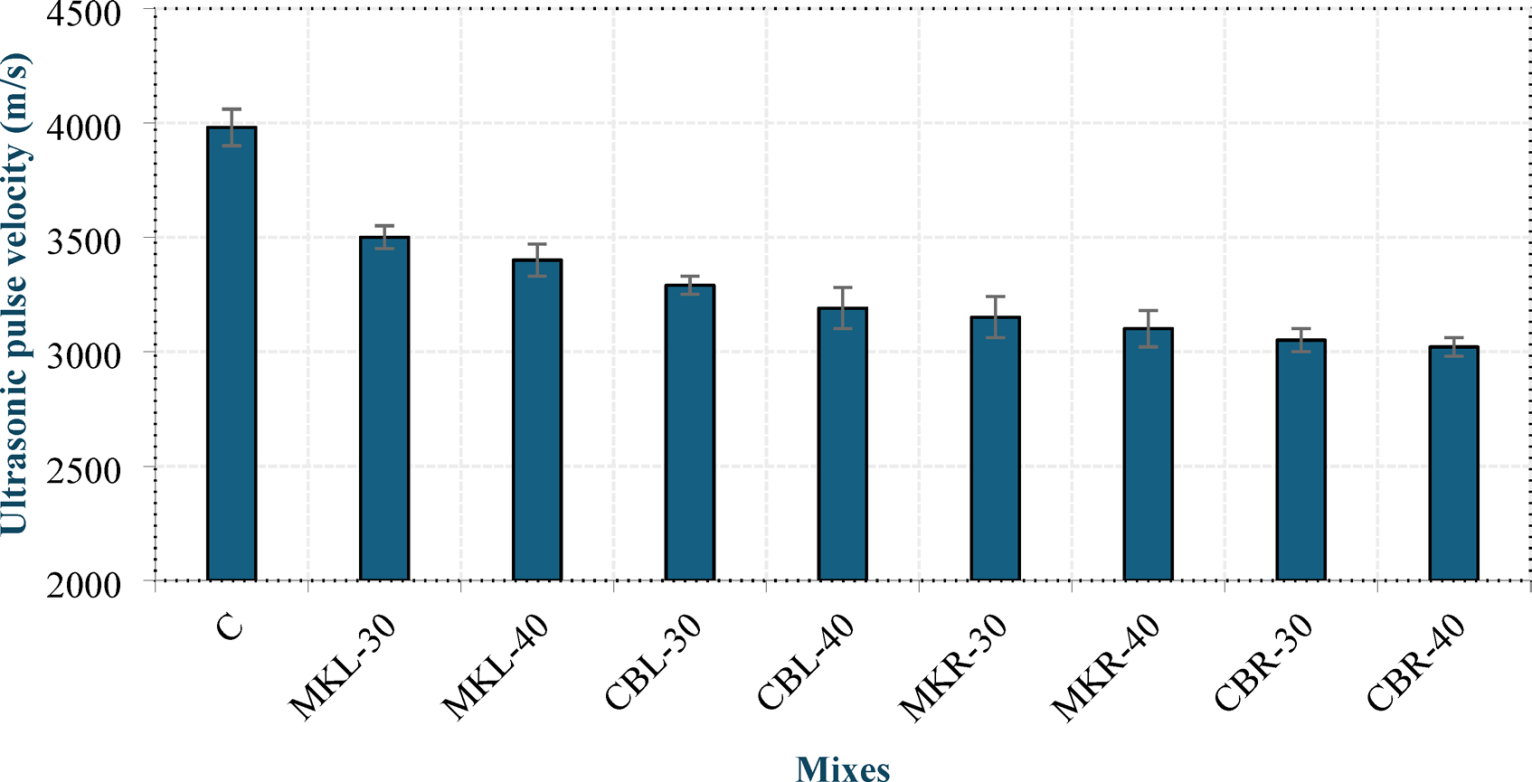
UPV of LC^3^ LWC at 28 days.

The UPV test is a non-destructive technique employed to evaluate the uniformity and overall quality of concrete. It determines the propagation velocity of ultrasonic waves through a material, which reflects its density and internal cohesion. Accordingly, the UPV can be indirectly utilized to estimate concrete strength, particularly compressive strength^43^. Figure 10presents the results of the UPV test on the produced concrete at 28 days. Mix C exhibited the highest UPV among all mixtures, which can be attributed to the absence of AEA, resulting in a denser and more homogeneous internal structure. Increasing the replacement level of MK or CBP from 30% to 40% led to a decrease in UPV values. In the case of MK, the UPV decreased by 2.86% in group L and 1.59% in group R, whereas CBP replacement caused reductions of 3.04% and 0.98% in groups L and R, respectively. This reduction in UPV is likely associated with a decreased formation of C-S-H and C-A-S-H gels, as the lower OPC content at higher replacement levels reduces the availability of CH required for the pozzolanic reaction. This explanation is consistent with the findings of Youssif et al^40^., who reported a decrease in C-S-H formation with increasing MK content.

**Fig. 11:**
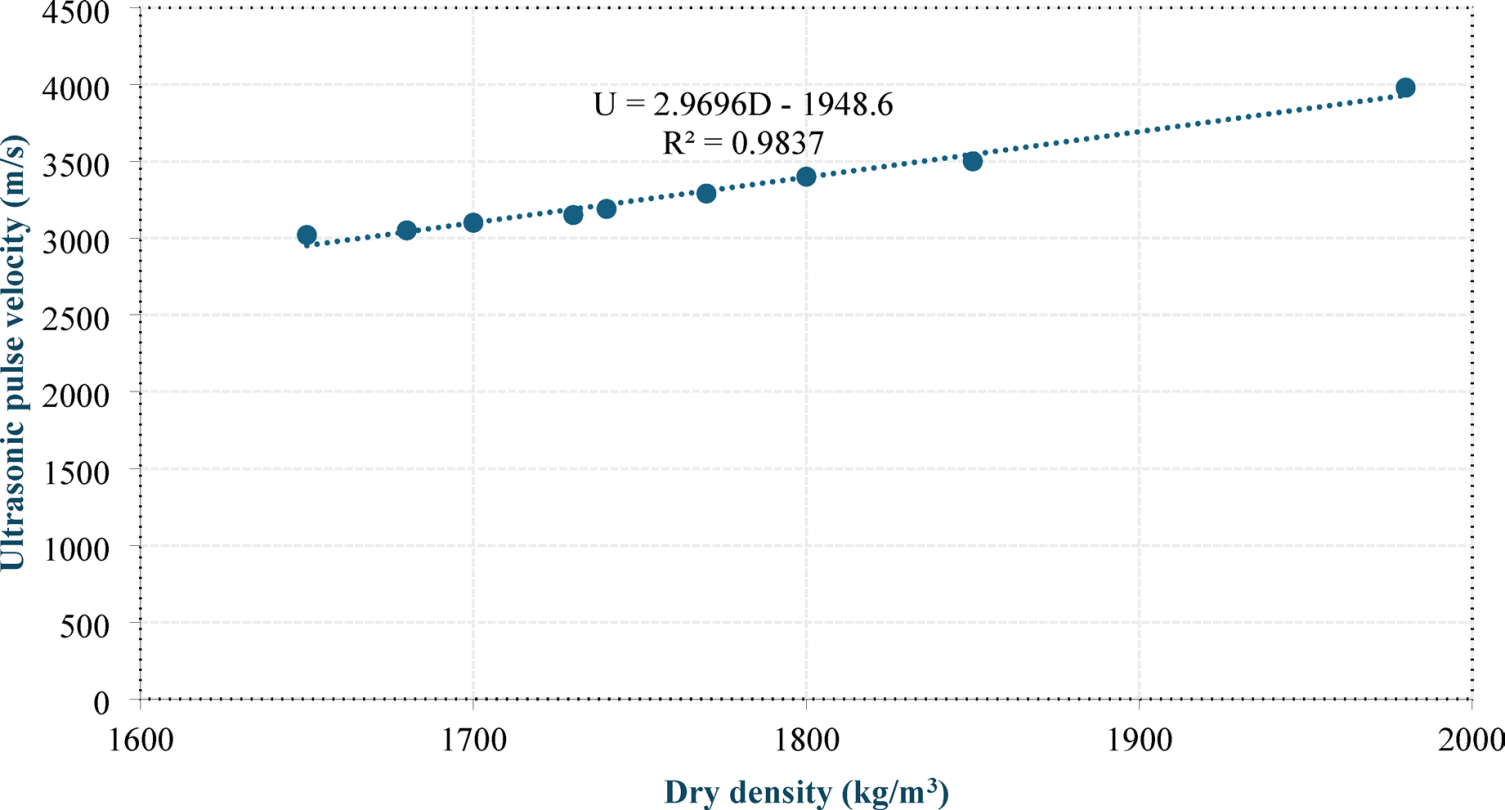
Correlation between dry density and UPV.

The incorporation of CBP resulted in lower UPV values than those of MK at equivalent replacement levels. Group L exhibited reductions of 6% and 6.18% at 30% and 40% replacement, respectively, whereas group R showed corresponding reductions of 3.17% and 2.58%, respectively. This behavior can be attributed to the inherently higher internal porosity of the CBP, which weakens the concrete matrix and hinders the transmission of ultrasonic waves, as sound propagates more rapidly through solid, dense materials than through porous materials. As shown in the figure, the mixes in group R presented lower UPV values than those in group L, with reductions ranging from 5.33% to 10%. This reduction can be attributed to the porous nature and irregular morphology of the RCP particles, which promote additional air entrapment within the matrix, whereas the filler effect of LSP enhances the particle packing efficiency, leading to a denser microstructure. These findings are consistent with the dry density results, where a similar trend was observed. Figure 11 shows the correlation between dry density and UPV, confirming the close relationship between the two parameters. Equation (6) presents the equation of the best-fit line passing through the data points.

6 $${\rm U = 2.9696 D - 1948. }$$

7 $${\rm C = 0.014\: U- 16.66}$$

Where U is the UPV (m/s) and D is the dry density (kg/m^3^).

#### Compressive strength

**Fig. 12:**
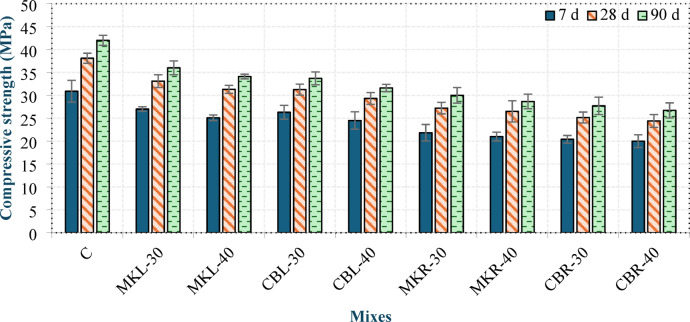
Compressive strength of LC^3^ LWC at 7, 28 and 90 days.

Figure 12 illustrates the compressive strengths of the produced concrete at 7, 28 and 90 days. At 28 days, the measured compressive strengths of all mixtures ranged from 24.4 to 38.1 MPa. It should be noted that the compressive strength values reported in this study were obtained from 70.5 mm cubes according to ASTM C109^32^. DIN EN 1045-1^15^ defines strength classes for lightweight concrete (e.g., LC 20/22) based on characteristic cylinder (150 × 300 mm) and standard cube (150 mm) strengths. While direct conversion between different specimen geometries can be complex and was not performed, the measured 28-day cube strengths for all LC³ mixtures ranged from 24.4 to 38.1 MPa. These values substantially exceed the minimum characteristic cube strength (22 MPa) required for the LC 20/22 class. Furthermore, all mixtures achieved dry densities below 1850 kg/m³, comfortably meeting the density criterion for structural lightweight concrete (< 2000 kg/m³). Therefore, the produced concrete can be reliably classified as structural LWC according to DIN EN 1045-1^15^.

Mix C achieved the highest compressive strength at 7, 28, and 90 days among all mixtures. Nevertheless, the other mixtures also fulfilled the structural requirements specified for LWC. The results shown in the figure clearly indicate that increasing the replacement level of either MK or CBP led to a slight reduction in the compressive strength of the concrete at all ages. At 40% MK, the compressive strength decreased by 7.04% and 3.83% at 7 days, 5.44% and 2.57% at 28 days, and 5.28% and 4.5% at 90 days in groups L and R, respectively, compared with that of the mixes containing 30% MK. Similarly, incorporating 40% CBP reduced the compressive strength by 6.84% and 1.96% at 7 days, 6.18% and 2.98% at 28 days, and 6.23% and 3.61% at 90 days in groups L and R, respectively, relative to their 30% CBP counterparts.

This reduction can be attributed to the diminished formation of C–S–H and C–A–S–H, resulting from the lower availability of CH necessary for the pozzolanic reaction due to the decreased OPC content at higher replacement levels. Atyia et al^14^. reported that the compressive strength of concrete decreases at all ages with increasing CBP content when the replacement level exceeds 20 wt%. The mixes incorporating CBP exhibited slightly lower compressive strengths than those containing MK at the same replacement level at all ages.

In group L, the reductions were 2.59%, 5.59% and 6.39% at 7, 28 and 90 days, respectively, for the 30% replacement and 2.39%, 6.33% and 7.33% at 7, 28 and 90 days, respectively, for the 40% replacement. In group R, the reductions reached 6.58%, 7.54% and 7.67% at 7, 28 and 90 days, respectively, for the 30% replacement and 4.76%, 7.92% and 6.81% at 7, 28 and 90 days, respectively, for the 40% replacement.

This can be attributed to the slightly lower pozzolanic activity for CBP than for MK, as discussed in the TGA and SAI results section. This may also be due to the high internal porosity of the CBP, which weakens the concrete matrix. As illustrated in the figure, the mixes in group R exhibited lower compressive strength values than those in group L, with reductions ranging from 16.33% to 22.43% at 7 days, 15.34% to 19.52% at 28 days, and 15.51% to 17.8% at 90 days. This reduction can be attributed to the porous structure and irregular morphology of the RCP particles, which facilitate additional air entrapment within the matrix.

In contrast, the superior performance of group L mixtures can be attributed to the well-documented filler effect of LSP, which enhances particle packing efficiency and contributes to a more compact matrix. This is supported by the lower porosity and higher density values observed for these mixtures (as shown in Fig. 9), which indicate a denser microstructure. These findings are consistent with the work of Wang et al^38^., who demonstrated the role of LSP in refining the pore structure of cementitious composites through its nucleation and filling effects.

In LC³ systems, bond performance is maintained through several mechanisms:

**First**, the pozzolanic reaction of MK and CBP consumes calcium hydroxide (CH) and promotes the formation of additional calcium-alumino-silicate-hydrate (C-A-S-H) gels. These gels densify the matrix and improve the interfacial transition zone (ITZ) by filling micro-voids and enhancing chemical bonding between the paste and aggregate surfaces^3,44^.

**Second**, the filler effect of LSP in group L mixtures improves particle packing and provides nucleation sites for hydration products, resulting in a denser ITZ with reduced porosity. This is supported by the lower water absorption and higher density values observed for these mixtures (Figs. 9 and 19), which indirectly indicate better bond quality^38^.

**Third**, for mixtures incorporating RCP and CBP, the bond performance is challenged by the porous nature and irregular morphology of these recycled materials. The adhered mortar on RCP particles and the internal porosity of CBP create a weaker ITZ, as evidenced by the higher porosity (13–18% increase) and lower compressive strength (15–20% reduction) in group R mixtures. This is consistent with observations by Xie et al^42^., who reported that the ITZ in recycled aggregate concrete is often the weakest link due to micro-cracks and porous remnants. The strength reduction is consistent with the findings of Ahmad et al^45^., who attributed such loss to the weak ITZ between recycled particles and cement paste in their comprehensive review on e-waste aggregates in concrete.

Despite these challenges, all LC³ mixtures met the structural requirements of DIN EN 1045-1^15^, indicating that the bond performance, while potentially inferior to the control, was sufficient to achieve the necessary load-bearing capacity. The continuous hydration and pozzolanic reactions within the LC³ matrix progressively densify the ITZ over time, as supported by the strength gain observed from 7 to 90 days (Fig. 12).

These findings are consistent with the SAI, dry density and UPV results. The mechanical performance of LC³ mixtures observed in this study aligns with the sustainability metrics reported by Kanagaraj et al^46^., who demonstrated that LC³ systems achieve superior cost-effectiveness and carbon efficiency while maintaining compressive strength comparable to conventional concrete. The 28-day compressive strengths achieved in our study (24.4–38.1 MPa) fall within the range suitable for structural applications, and when combined with the reduced clinker content (up to 60%), these mixtures offer a balance between mechanical performance and environmental sustainability consistent with the findings of Kanagaraj et al^46^..

**Fig. 13:**
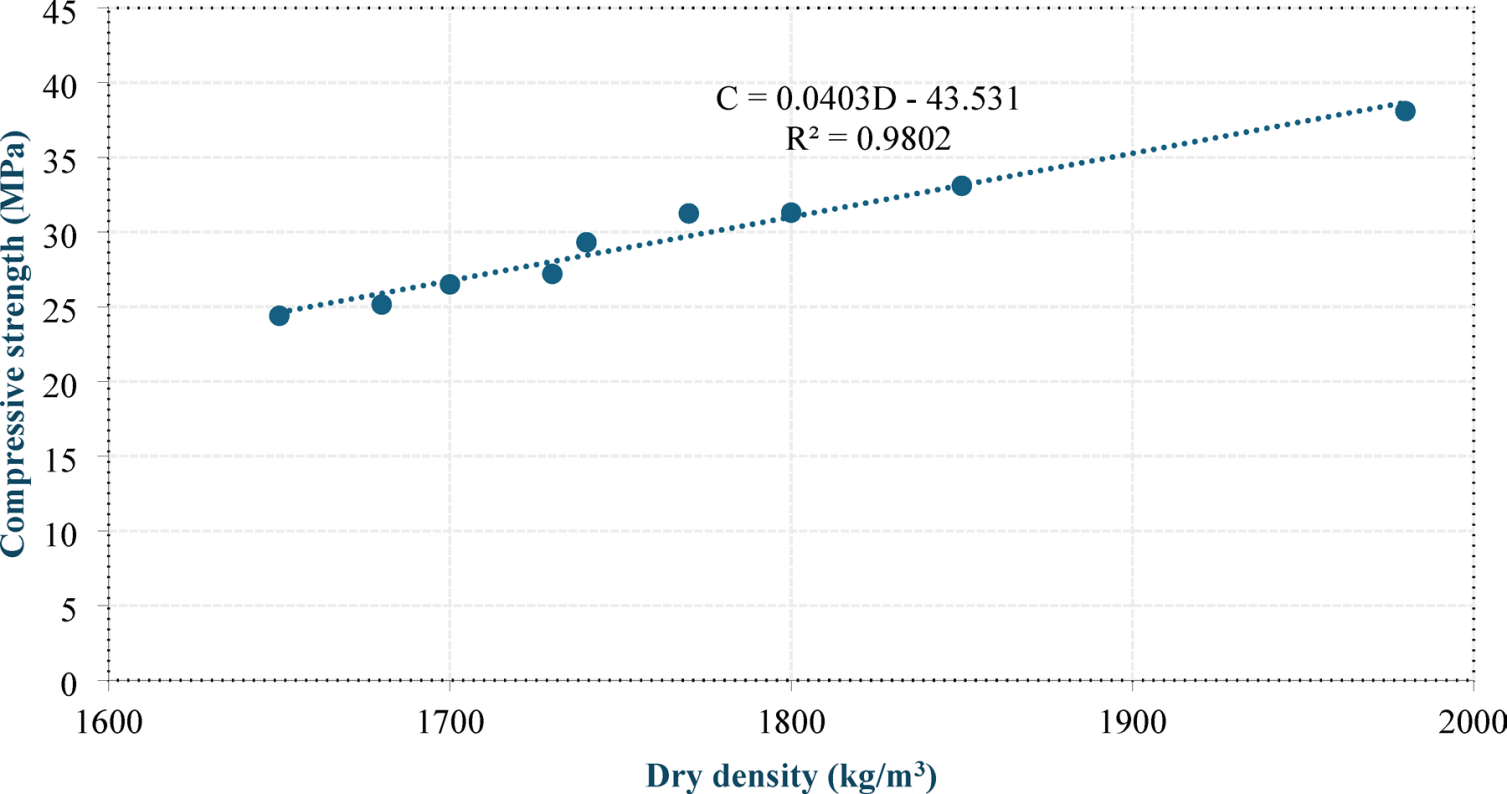
Correlation between compressive strength and dry density.

**Fig. 14:**
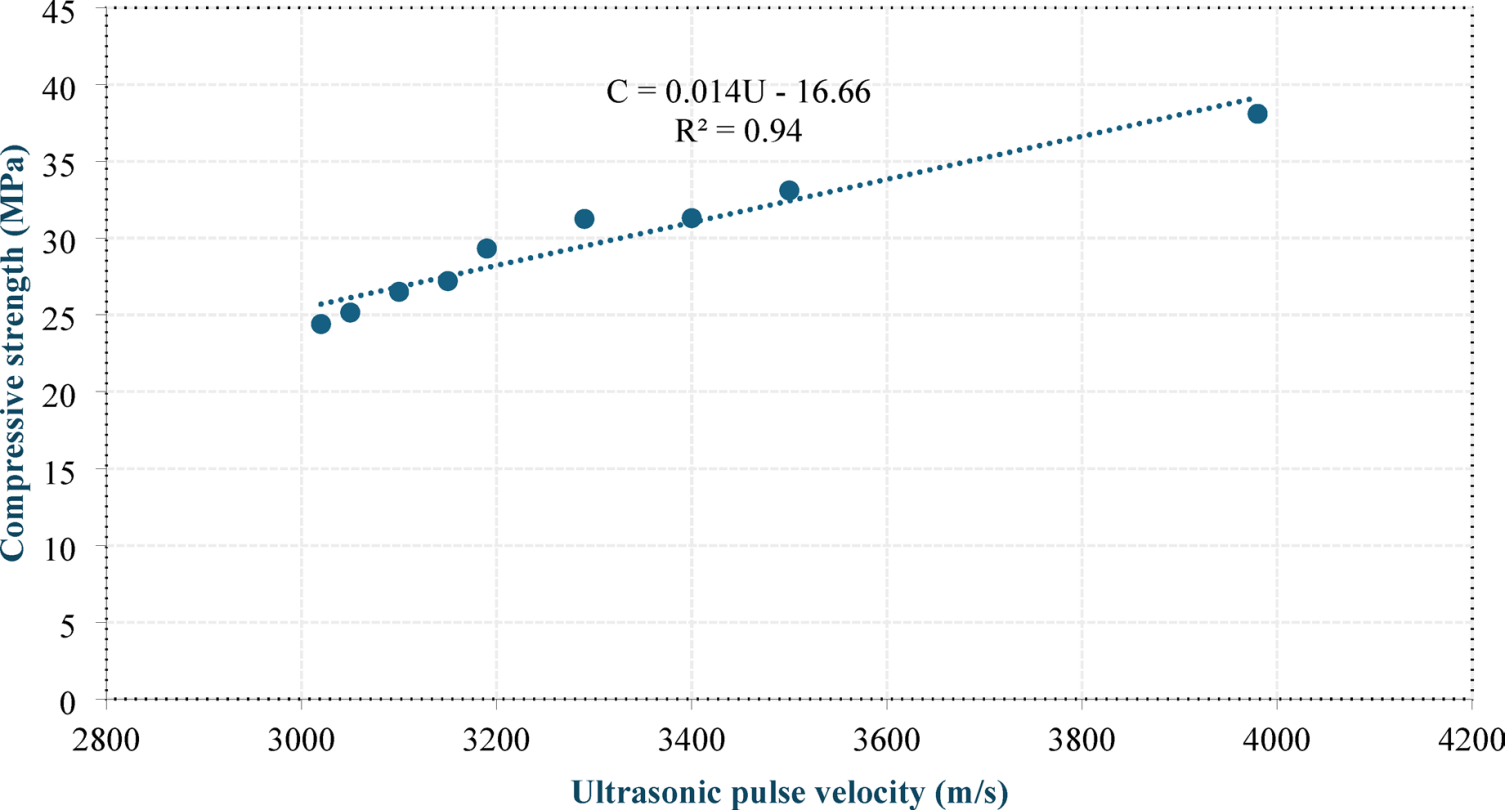
Correlation between the compressive strength and UPV.

Figures 13 and 14 show the correlations between the 28-day compressive strength and dry density and between the 28-day compressive strength and UPV, respectively. Equation (7) and Eq. (8) present the equations of the best-fit lines passing through the data points.

8 $${\rm C = 0.0403 D-43.531}$$

9 $${\rm C = 0.014\: U- 16.66}$$

where C is the 28-day compressive strength (MPa), D is the dry density (kg/m^3^), and U is the UPV (m/s).

Table 4 shows a direct comparison between the current study and the most relevant previous studies that used recycled materials in LC³ systems.

**Table 4:** Comparison of the current study’s results with previous research on LC³ systems incorporating recycled materials.

Study	Binder Type	Aggregate Type	Specimen Type	Compressive Strength (MPa)	Dry Density (kg/m³)
Tang et al^8^.	LC³ (with RCP)	Natural sand	Mortar/Paste	-	-
Marangu^9^	LC³ (with brick powder)	-	Paste	-	-
Alghamdi et al^10^.	LC³	Autoclaved aerated concrete waste	Mortar	13–29.8	≤ 1300
**Current Study**	**LC³ (CBP + RCP)**	**Crushed brick (fine + coarse)**	**Structural LWC (cubes)**	**24.4–38.1**	**1650–1850**

As shown in Table 4, while previous research has explored individual aspects of recycled materials in LC³, none have achieved the integrated approach presented in this work, which combines dual waste-based binders with lightweight aggregates to produce concrete that meets all structural requirements. This comparison shows the most important improvements made in this study, especially the fact that it was able to make a fully waste-based structural lightweight concrete.

#### Elevated temperature exposure

##### Residual mass

**Fig. 15:**
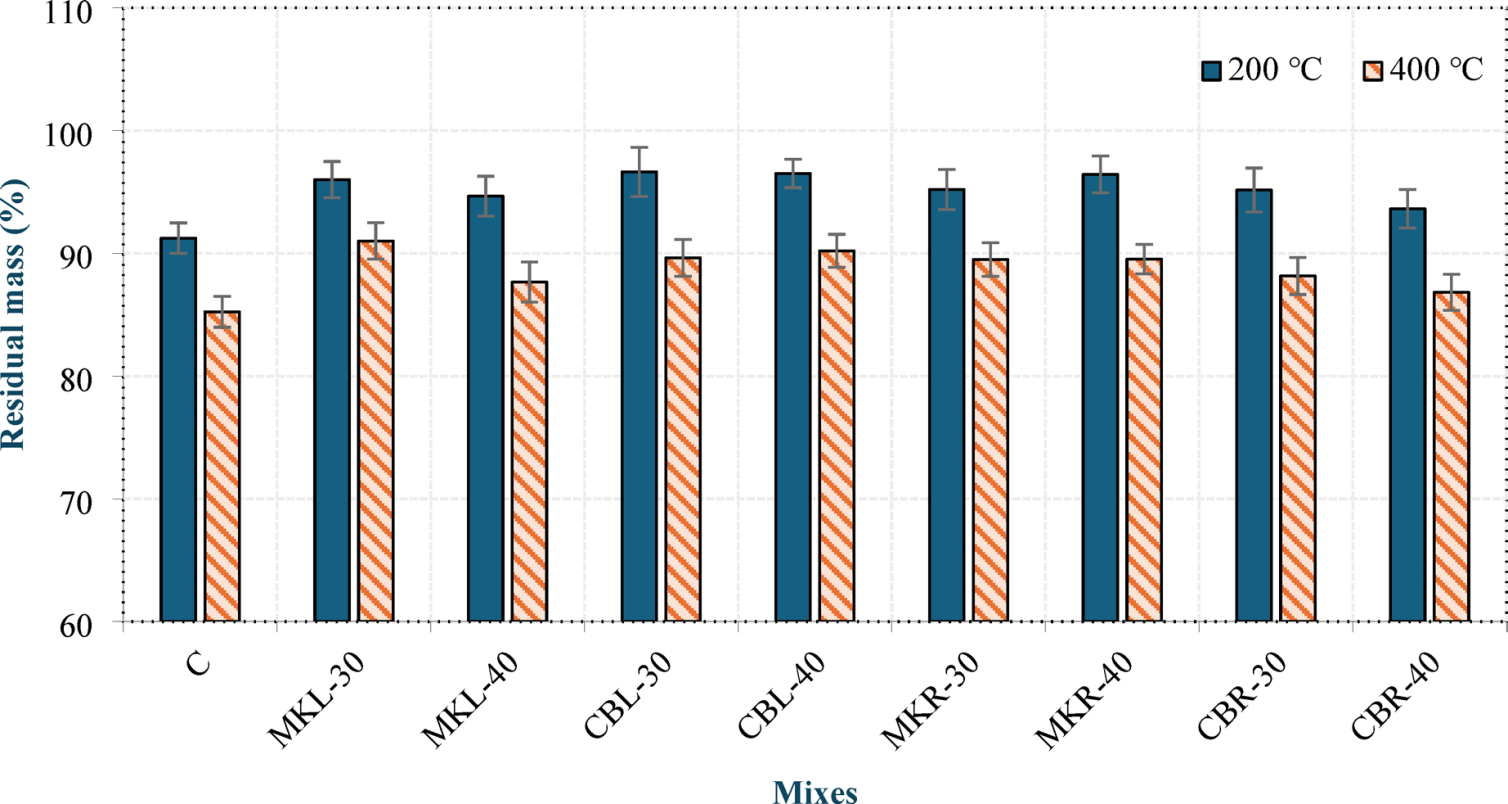
Relative residual mass of the concrete specimens exposed to 200 °C and 400 °C at 28 days.

Figure 15 illustrates the relative residual mass of the concrete specimens after exposure to 200 °C and 400 °C at 28 days. The relative residual mass is the ratio of the mass retained after heating to the original mass of the same specimens measured at room temperature. It is evident that the residual mass decreased with increasing temperature for all mixtures. At 200 °C, the relative residual mass of mix C was 91.24%, while it ranged from 94.65% to 96.63% in group L and from 93.63% to 96.43% in group R. The decrease in mass at 200 °C was due to the evaporation of free and physically bound water, leading to the formation of air voids and pore structure^47^. At 400 °C, the relative residual mass of mixture C was 85.24%, whereas it ranged from 87.65% to 91% in group L and 86.83% to 89.53% in group R. The decrease in mass at 400 °C is attributed to the release of chemically bound water from C–S–H and C–A–S–H, which generally occurs at temperatures up to 400 °C, leading to the development of greater porosity within the matrix^33^. C–S–H and C–A–S–H start very slow dehydration above 100 °C and increase with increasing temperature. The residual mass of mixture C was lower than that of all the other mixtures at 200 °C and 400 °C, indicating that the LC^3^LWC has good resistance to heat at moderate temperatures, which is in agreement with the findings of Gunjal et al^47^..

##### Residual compressive strength

**Fig. 16:**
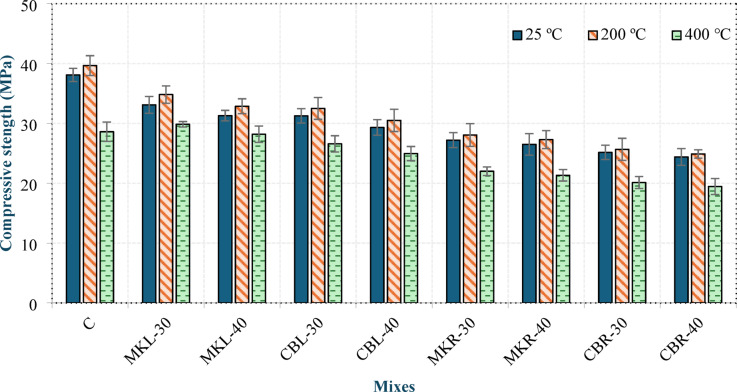
Residual compressive strengths of the concrete specimens exposed to 200 °C and 400 °C at 28 days.

The compressive strengths of the concrete specimens at ambient temperature and after exposure to 200 °C and 400 °C at 28 days are presented in Fig. 16. At 200 °C, the compressive strength of all mixtures slightly increased compared to the unheated specimens. The increase in the compressive strength of mix C was 4.1%, whereas it ranged from 4.01% to 5.2% in group L and from 2.03% to 3.13% in group R. The increase in the compressive strength observed at 200 °C can be attributed to the continued hydration of unhydrated cementitious particles facilitated by the presence of steam. Owing to its higher diffusivity than liquid water, steam penetrates more effectively into the matrix, generating an “internal autoclave effect” that promotes the formation of additional hydration products^33^. The entrained air voids in the LWC preserve moisture during early heating, facilitating continued hydration and temporary strength gain at 200 °C. At 400 °C, the compressive strength of all mixtures significantly decreased compared to the unheated specimens. The decrease in the compressive strength of mix C was 24.9%, whereas it ranged from 9.8% to 14.93% in group L and from 19.1% to 20.3% in group R. The reduction in the compressive strength at 400 °C was mainly due to the pronounced release of chemically bound water from C–S–H and C–A–S–H at this stage, which increased the porosity and weakened the matrix, thereby lowering the compressive strength of the concrete^33^. Mix C exhibited the greatest reduction in compressive strength among all mixtures at 400 °C, proving that the LC^3^-based LWC has better resistance to moderate thermal exposure. Compared with the L mixes, the R mixes presented a greater reduction in compressive strength at 400 °C. This can be attributed to the lower SAI value of RCP, which constrains its high-temperature efficiency compared with that of LSP. Furthermore, the irregular morphology of RCP particles may facilitate the formation of interfacial microcracks under thermal exposure, as has been observed in similar studies on recycled aggregates^16,42^, contributing to the greater strength deterioration in group R mixtures.

##### Performance of CDW-based mixtures under elevated temperatures

The behavior of mixtures incorporating construction and demolition waste (CDW) under elevated temperatures exhibited distinct characteristics compared to the control and conventional LC³ mixtures. As shown in Figs. 15 and 16, all CDW-based mixtures demonstrated acceptable thermal stability, retaining over 80% of their compressive strength at 400 °C, which meets the general requirements for structural concrete under moderate thermal exposure.

For CBP-based mixtures (CBL-30, CBL-40, CBR-30, CBR-40), the residual strength at 200 °C was comparable to that of MK-based mixtures, with increases ranging from 2.03% to 5.2%. However, at 400 °C, CBP-based mixtures showed slightly higher strength reduction (approximately 14–20%) compared to their MK counterparts. This can be attributed to the inherently higher porosity of CBP particles (as discussed in Sect. 4.2.2), which may facilitate heat-induced micro-cracking and accelerate the dehydration of hydration products within the ITZ^14,42^.

For RCP-based mixtures (group R), the performance under elevated temperatures was notably lower than that of LSP-based mixtures (group L). At 400 °C, RCP-based mixtures experienced compressive strength reductions of 19.1–20.3%, compared to 9.8–14.93% for LSP-based mixtures. This pronounced deterioration can be explained by several factors:

- The porous structure of RCP particles, including adhered mortar, which increases the susceptibility to thermal cracking^42^.
- The absence of the thermally stable filler effect provided by LSP, which in group L mixtures helped maintain matrix density and mitigate damage^38^.
- The irregular morphology of RCP particles, which may create a weaker ITZ that is more vulnerable to thermal stresses, as observed in similar studies on recycled aggregate concrete^16^.

Despite these reductions, all CDW-based mixtures maintained residual compressive strengths above 19 MPa at 400 °C, which remains within the acceptable range for lightweight concrete applications where moderate heat exposure is expected. The results indicate that while CDW-based LC³ systems are suitable for applications involving temperatures up to 200 °C, their performance at 400 °C may require additional optimization for critical structural elements.

These findings are consistent with previous research by Gunjal et al^47^. and Cao et al^33^., who reported that the thermal stability of LC³ systems is primarily governed by the type of supplementary cementitious materials and the porosity of the aggregate phase. The inclusion of CDW materials, while environmentally beneficial, introduces additional porosity that can influence thermal behavior, highlighting the need for careful mix design when targeting specific thermal exposure classes.

#### Magnesium sulfate exposure

##### Mass change

**Fig. 17:**
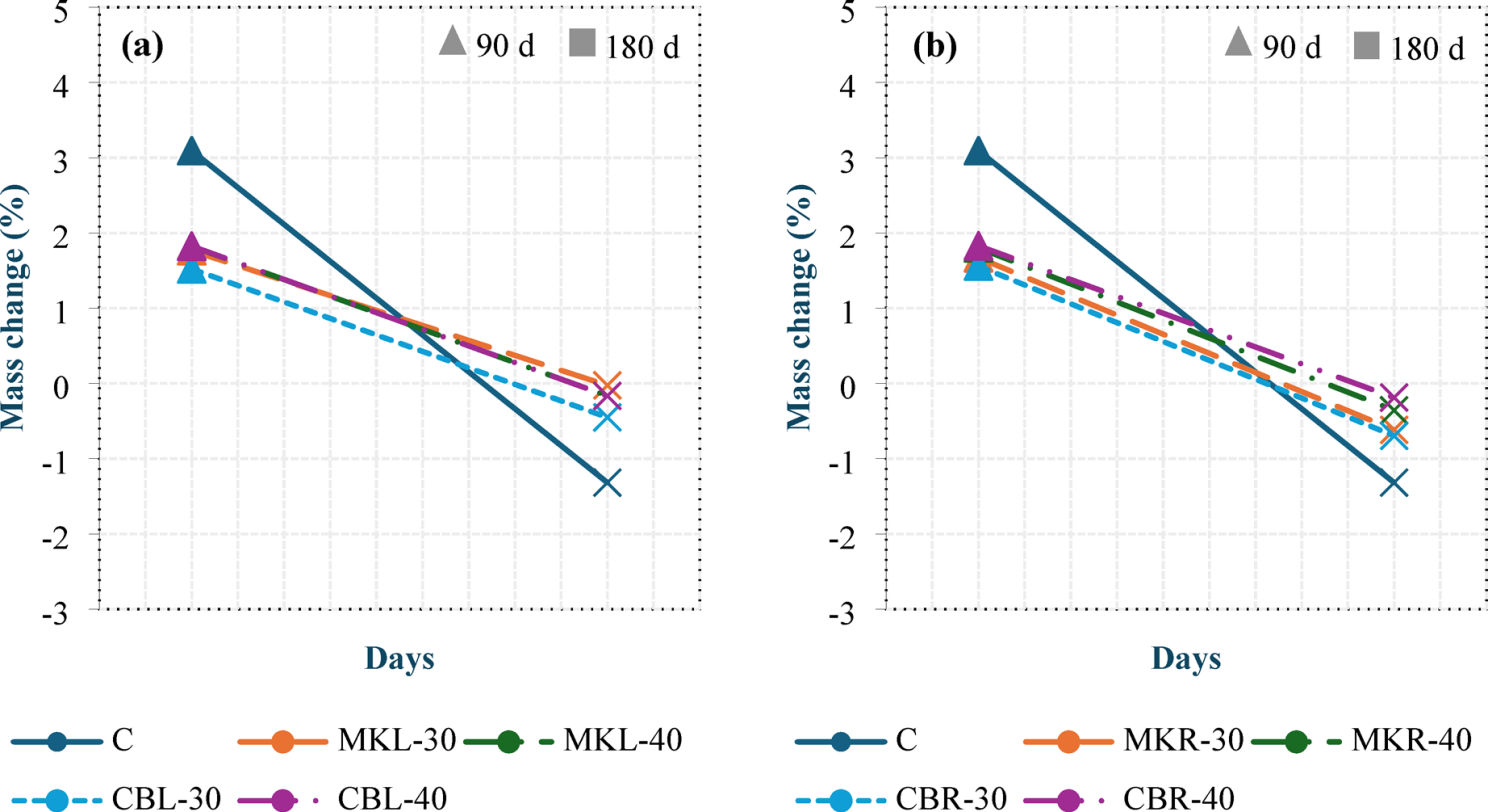
Mass change of group L (a) and group R (b) after 90 and 180 days of magnesium sulfate exposure.

Figure 17 shows the mass change of all concrete specimens after 90 and 180 days of magnesium sulfate exposure. Mix C resulted in the greatest mass gain among all mixtures at 90 days, with a 3.09% increase relative to its initial mass before exposure. In comparison, the mass gain ranged between 1.52% and 1.83% in group L and between 1.56% and 1.83% in group R. The mass increase at 90 days may be due to the formation of ettringite and gypsum, which are produced from the reactions of sulfate with alumina and CH, respectively. The greater mass gain of mix C than the other mixtures can be explained by its relatively larger amount of CH, which reacts with sulfate ions to produce additional gypsum. At 180 days, a mass reduction was observed in all mixtures. Mix C resulted in the greatest mass loss, with a decrease of 1.32%, whereas the reduction ranged between 0.02% and 0.45% in group L and between 0.19% and 0.7% in group R. This decrease in mass can be attributed to the disruptive expansion caused by the formation of gypsum and ettringite, which led to deterioration and subsequent material loss. The more pronounced loss in mix C is explained by its higher CH content, which promotes excessive gypsum formation, in addition to the absence of AEA, unlike the other mixtures, which were able to accommodate internal expansion better. These results indicate that the LC^3^LWC possesses good resistance to sulfate attack, which is consistent with the findings reported by Shi et al^44^., which indicated that LC^3^ has superior resistance to sulfate attack for up to 396 days.

## Residual compressive strength

**Fig. 18:**
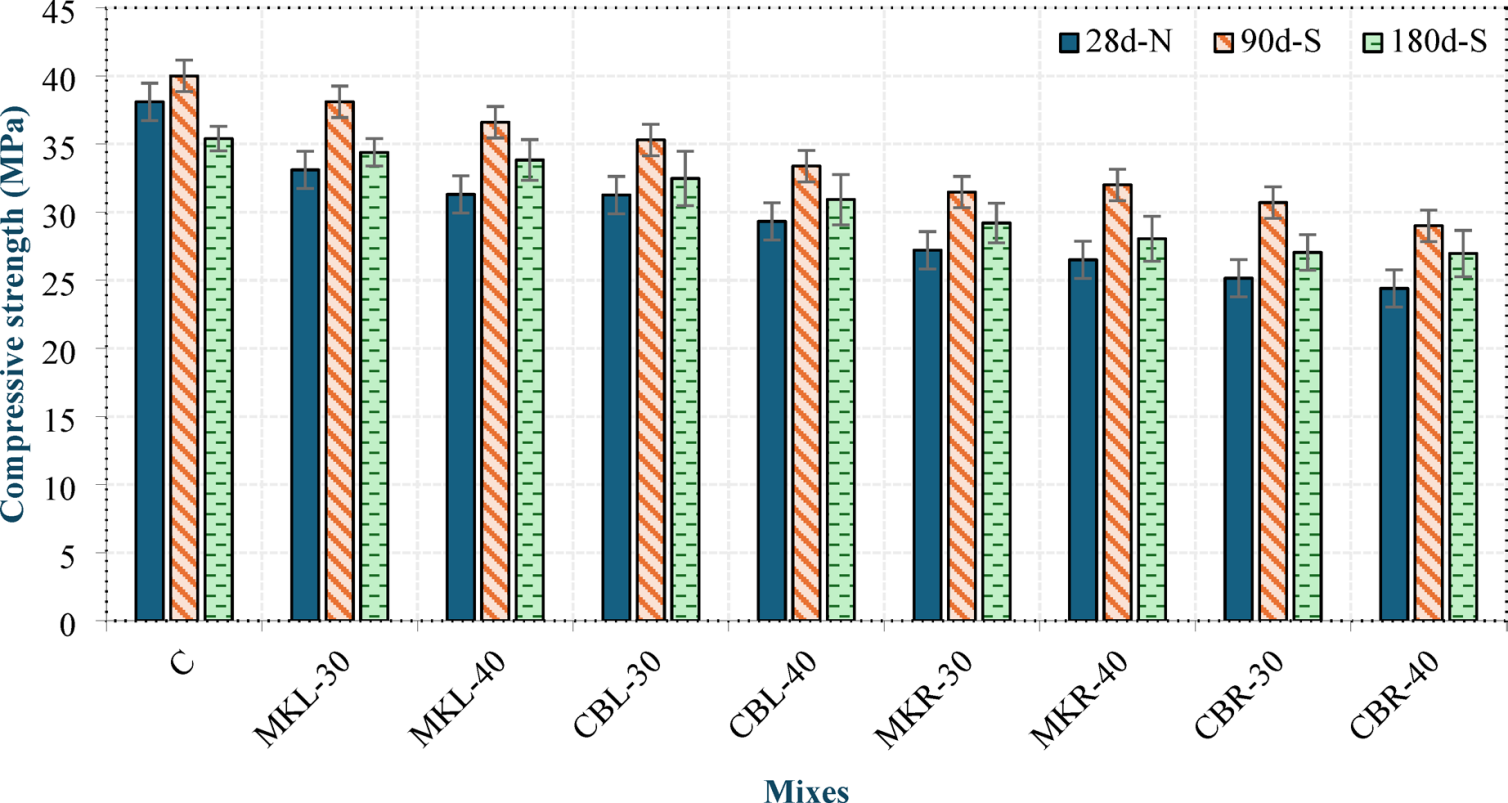
Compressive strength of the specimens under normal conditions (28 d-N) and after 90 (90 d-S) and 180 (180 d-S) days of magnesium sulfate exposure.

The compressive strengths of all concrete specimens under normal conditions (28 d-N), as well as after 90 (90 d-S) and 180 (180 d-S) days of exposure to magnesium sulfate, are shown in Fig. 18. It is evident that the compressive strength of all mixtures improved at 90 days compared to the specimens under normal conditions; however, the increase in the compressive strength of mixture C was lower than that of the other mixtures. The increase in compressive strength at 90 days can be attributed to the continued hydration of previously unhydrated particles, as well as the formation of ettringite and gypsum within the pore structure, which temporarily fill voids and densify the matrix. At 180 days, the compressive strengths of groups L and R decreased compared with their values at 90 days but remained higher than those under normal conditions, whereas mix C exhibited a reduction below the strengths of its corresponding specimens under normal conditions. Mix C resulted in a 7.11% lower strength relative to normal conditions. In contrast, the compressive strength of group L exceeded that of the normal-condition specimens by between 3.89% and 8.07%, whereas that of group R exceeded them by between 5.81% and 10.49%. The reduction in compressive strength at 180 days relative to that at 90 days can be attributed to the expansive formation of ettringite and gypsum, which generated micro-cracks and consequently weakened the matrix. However, in groups L and R, this deterioration was not sufficient to reduce the strength below that of the specimens under normal conditions. This may also be due to the formation of magnesium silicate hydrate (M-S-H), resulting from the reaction between sulfate ions and C-S-H, which does not contribute to strength development. In contrast, the pronounced strength loss observed in mix C compared with its normal-condition specimens can be explained by its higher CH content, which promotes excessive gypsum formation and thereby increases microcracking. Shi et al^44^. reported that the lower tricalcium aluminate content in the LC³ system than in OPC reduces the potential for expansive ettringite formation, thereby enhancing sulfate resistance. Furthermore, the absence of AEA in mixture C, unlike in the other mixtures, limited its ability to accommodate internal expansion. These findings are consistent with the mass change results and further confirm that the LC^3^ LWC exhibits high resistance to sulfate attack.

## Water absorption and porosity

**Fig. 19:**
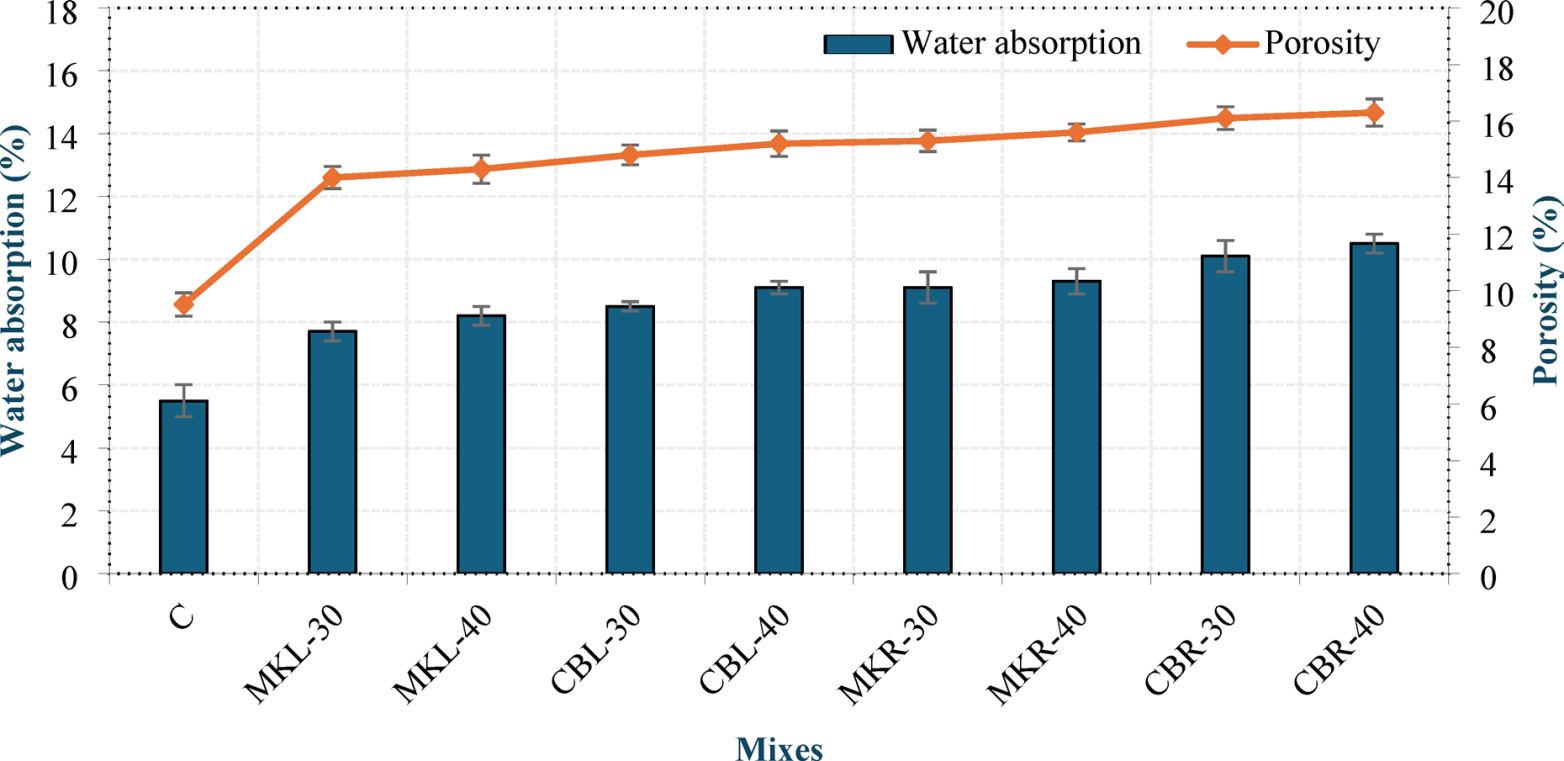
Water absorption and porosity of LC^3^ LWC at 28 days.

The porosity represents the volume fraction of voids within the hardened concrete matrix, which directly influences its durability and permeability. Water absorption refers to the capacity of concrete to absorb and retain water through these pores. Figure 19 presents the concrete water absorption and porosity at 28 days. A direct correlation was observed between water absorption and apparent porosity. Mix C resulted in the lowest values of water absorption and porosity among all mixtures, which is attributed to the absence of AEA and the consequent development of a denser, more homogeneous internal structure. Increasing the replacement level of MK or CBP from 30% to 40% resulted in an increase in both water absorption and porosity. For MK, water absorption increased by 6.49% in group L and 3.26% in group R, whereas CBP incorporation led to higher increments of 7.06% and 3.96% in groups L and R, respectively. This trend can be related to the reduced formation of C–S–H gel due to the lower OPC content at higher replacement levels. At equivalent replacement percentages, CBP consistently produced greater water absorption and porosity than MK, with group L showing increases in water absorption of 10.39% and 10.98% at 30% and 40% replacement and group R exhibiting 9.78% and 10.53% at the same levels. This behavior is mainly associated with the intrinsically higher porosity of CBP. Furthermore, group R consistently presented higher water absorption and porosity values compared to group L, with increases in water absorption ranging from 13.41% to 18.18%.

This can be explained by the intrinsically porous structure and irregular particle morphology of RCP, which tend to entrap air within the matrix, as well as the absence of the well-established filler effect provided by LSP. The filler effect of LSP, which improves particle packing and promotes a denser microstructure, has been thoroughly documented in the literature^38^. These observations are in line with the results obtained from dry density, UPV, and compressive strength tests.

## Conclusions

This study successfully established the viability of producing sustainable structural lightweight concrete (LWC) by incorporating construction and demolition waste as both binder components and aggregates in limestone calcined clay cement (LC³) systems. The key findings are summarized as follows:

- **Crushed brick powder (CBP)** proved to be a suitable pozzolanic alternative to metakaolin (MK) in LC³-based LWC, demonstrating comparable performance with only a marginal 5–8% reduction in 28-day compressive strength and a 10–12% increase in water absorption, while fully satisfying the structural LWC requirements of DIN EN 1045-1 (density < 1850 kg/m³, strength > 24 MPa).
- **Recycled concrete powder (RCP)** effectively substituted limestone powder (LSP) in LC³ systems. Although this replacement resulted in a more pronounced decrease in 28-day compressive strength (15–20%) and an increase in water absorption (13–18%), all RCP-based mixtures met structural criteria and offered significant environmental benefits through resource conservation and waste valorization.
- **A clinker reduction of up to 60%** was achieved without compromising mechanical performance, highlighting the potential of LC³ technology for producing low-carbon, high-performance, and sustainable concrete.
- **Performance under diverse exposure conditions**:
- **Ambient conditions**: All mixtures met DIN EN 1045-1 requirements, with 28-day compressive strengths of 24.4–38.1 MPa and dry densities of 1650–1850 kg/m³, confirming suitability for general structural applications including reinforced concrete elements and precast components.
- **Moderate thermal exposure (up to 400 °C)**: All LC³ mixtures retained over 80% of their compressive strength, outperforming the OPC control. CBP-based mixtures exhibited thermal stability comparable to MK-based mixtures, while RCP-based mixtures showed slightly higher strength loss (19–20%) yet remained suitable for non-structural applications such as insulating layers or non-load-bearing walls.
- **Severe sulfate exposure (180 days)**: All LC³ systems demonstrated superior durability, with mass loss below 0.7% and compressive strength retention exceeding 95% of normal-condition values, confirming their appropriateness for aggressive environments including foundations, sewage systems, marine applications, and soil-contact elements.
- **The integrated use of waste-derived aggregates (crushed brick) and binders (CBP and RCP)** successfully produced concrete that balances structural performance with significant environmental advantages, including waste valorization, reduced natural resource depletion, and a lower carbon footprint.

These findings confirm that LC³-based lightweight concrete incorporating C&D waste is a versatile and sustainable solution suitable for a wide range of exposure conditions, contributing to both structural integrity and environmental sustainability.

## Current limitations and future perspectives

Although this study yielded promising results, several limitations should be acknowledged. The durability evaluation was confined to 180 days of exposure to magnesium sulfate, leaving the long-term deterioration mechanisms beyond this period unexamined. Likewise, the assessment of elevated temperature resistance was restricted to moderate exposure levels (200 °C and 400 °C), without considering the material’s behavior at higher temperatures.

Future research should therefore extend the sulfate exposure period to 365 days to obtain a more comprehensive understanding of the long-term performance of LC³ concrete. In addition, examining the behavior at temperatures above 400 °C would provide valuable insight into its thermal stability. Further investigations are also encouraged to perform detailed microstructural characterization—both at ambient conditions and after thermal exposure—using advanced analytical tools such as SEM, MIP, XRD, and µ-CT.

A comprehensive sustainability assessment framework, like that employed by Banar et al^48^., would further validate the environmental and economic benefits of the developed LC³ mixtures. Their study demonstrated that SCM-based concretes, particularly those containing metakaolin, achieved significant reductions in CO₂ emissions per MPa per service year (up to 94%) while maintaining superior technical performance. Adopting such integrated metrics, such as the Building Material Sustainability Potential (BMSP) index, could provide a holistic comparison of LC³-based mixtures with conventional concretes in future investigations.

Expanding the scope of mechanical testing to include flexural and tensile strength, as well as exploring the application of LC³ systems in other types of concrete, would contribute to a more holistic assessment of their structural and sustainability potential.

## Data Availability

All the data used are provided within the manuscript.

## Abbreviations

AEA: Air-entraining agent
C&D: Construction and demolition
C-A-S-H: Calcium alumino-silicate hydrate
CB: Crushed bricks
CBCA: Crushed brick coarse aggregates
CBFA: Crushed brick fine aggregates
CBP: Crushed brick powder
CH: Calcium hydroxide
C-S-H: Calcium silicate hydrate
LC^3^: Limestone calcined clay cement
LOI: Loss on ignition
LSP: Limestone powder
LWC: Lightweight concrete
MK: Metakaolin
M-S-H: Magnesium silicate hydrate
OPC: Ordinary Portland cement
RCP: Recycled concrete powder
SAI: Strength activity index
SCMs: Supplemental cementitious materials
SP: Superplasticizer
SSD: Saturated surface-dry
TGA: Thermogravimetric analysis
UPV: Ultrasonic pulse velocity
w/b: Water-to-binder ratio
w/c: Water-to-cement ratio
XRD: X-ray diffraction analysis
